# Structure, disorder, and dynamics in task-trained recurrent neural circuits

**DOI:** 10.64898/2026.03.02.708943

**Published:** 2026-05-18

**Authors:** David G. Clark, Blake Bordelon, Jacob A. Zavatone-Veth, Cengiz Pehlevan

**Affiliations:** 1 Kempner Institute for the Study of Natural and Artificial Intelligence, Harvard University, Cambridge, MA, USA; 2 Center of Mathematical Sciences and Applications, Harvard University, Cambridge, MA, USA; 3 Society of Fellows, Harvard University, Cambridge, MA, USA; 4 Center for Brain Science, Harvard University, Cambridge, MA, USA; 5 John A. Paulson School of Engineering and Applied Sciences, Harvard University, Cambridge, MA, USA

## Abstract

Across many brain areas, neurons produce heterogeneous, seemingly disordered responses. Yet such circuits cannot be purely random, since they must possess some structure to generate the representations and computations underlying behavior. How much structure is present in recurrent connectivity relative to disorder, and how the interaction between the two shapes population dynamics and single-neuron responses, remain incompletely understood. Recurrent neural networks trained to perform tasks have become a leading model of such circuits, but conventional training yields a single point in a vast space of task-compatible solutions, with no systematic way to explore this space and no theory of how internal representations vary within it. Without such a theory, the questions above cannot be addressed, and comparisons between trained networks and neural data are difficult to interpret. Here, we introduce a control parameter that governs the degree to which learning reshapes recurrent connectivity, interpolating between a reservoir regime and one in which recurrent weights are restructured by learning to produce task-relevant internal representations. Varying this parameter generates a family of task-compatible solutions whose internal dynamics differ in a controlled and interpretable way. We derive a dynamical mean-field theory showing that, while population-level dynamics converge to a deterministic limit, individual neurons are driven by independent samples from a single-neuron input-current distribution. When connectivity is random, this distribution is Gaussian. Recurrent restructuring drives it toward task-dependent, non-Gaussian forms. In linear networks, restructuring amplifies task-relevant frequencies. In nonlinear networks, it drives a phase transition from chaotic, high-dimensional activity to ordered, low-dimensional dynamics that generalize temporally beyond the training period. We apply the theory to a reaching task in which a recurrent network must reproduce macaque muscle activity, and find that optimally matching simultaneous motor-cortex recordings requires only a small degree of restructuring, with learned structure coexisting with random heterogeneity. These results suggest a broader picture in which large recurrent circuits are largely random but contain, to varying degrees, structured recurrent connectivity sufficient for generalizable, task-relevant representations.^1^

## 1 Introduction

Neurons in early sensory areas often exhibit orderly response properties. In primary visual cortex, for example, individual neurons respond selectively to oriented edges [1], with the population tiling the space of orientations in an organized fashion [2]. Similarly orderly tuning has been observed in auditory and somatosensory cortices [3]. Inspired by these successes, early work in motor neuroscience sought analogous structure in motor cortical responses, seeming to find cells in primary motor cortex with cosine-like tuning to the direction of arm reaches [4].

Subsequent experiments and analyses indicated that, during reaching, motor cortical neurons produce multiphasic, heterogeneous, and highly dynamic responses that resist description via simple tuning curves [5, 6]. Such complexity appears to be the rule rather than the exception across large mammalian circuits. Head-direction cells, despite being organized around a one-dimensional variable, show striking response heterogeneity [7]. Hippocampal place fields are diverse and spatially irregular [8]. Neurons in prefrontal cortex carry mixed, condition-dependent signals during cognitive tasks [9]. Premotor neurons show richly diverse temporal responses during movement preparation and execution [10–12]. This pervasive heterogeneity may lead one to think that neural circuits are largely random, an idea with empirical support in systems such as the insect mushroom body and mammalian piriform cortex, where projections appear to be substantially unstructured [13–15].

However, random connectivity on its own cannot be the full story. Recurrent circuits with random connectivity, called *reservoir* networks, can generate feature selectivity [16] and perform pattern separation [17], but they generally produce high-dimensional chaotic activity [18, 19]. Such activity seems clearly at odds with the structured, low-dimensional population-level dynamics observed in motor cortex [6], in areas underlying cognitive tasks, and in other circuits [20]. Structured recurrent connectivity is also beneficial for generating representations that support generalization and compositionality [21, 22]; we analyze generalization in a simplified scenario below. Neural circuits thus likely contain both a degree of disorder in their connectivity, perhaps due to stochastic wiring rules [23], and some learned structure that generates task-relevant representations. How much structure is present relative to disorder, and how the interaction between them shapes population-level dynamics and single-neuron responses, remain incompletely understood.

Addressing these questions in a circuit model requires a way to endow the network with task-relevant connectivity. One approach, which has proven amenable to theoretical analysis, is to specify the desired neuronal representation and construct connectivity that supports it [24–26]. This allows one to study the resulting, generically disordered connectivity and network dynamics, but the neuronal responses themselves are fixed *a priori*. A more ambitious approach is task training, in which the representation is not imposed but learned, with the learning signal provided by asking the network to perform an experimentally relevant task. When optimized to perform motor, cognitive, or sensory tasks, such networks often develop neuronal responses and population-level dynamics that resemble those found in neural recordings [9–11, 22, 27, 28], allowing such comparisons to inform our understanding of the circuit at hand.

Despite the ubiquity of task-trained RNNs in systems neuroscience, we lack a theory of the representations these networks learn. Conventional training yields a single point in a vast space of task-compatible solutions, with no systematic way to explore how representations vary along the order-disorder axis and no theory of what governs this variation. This has two consequences. First, the questions of structure and disorder posed above cannot be addressed in a principled way. Second, comparisons between trained RNNs and neural data are difficult to interpret. Ideally, one would have a controllable parameter that generates diverse task-compatible solutions, paired with a theory of how that parameter shapes internal representations, so that when a particular solution matches neural data one can draw specific conclusions about the underlying circuit (we return to this point in the Discussion). A related practical issue is that, without such a theory, it is unclear under what conditions population-level dynamics are consistent across training runs and network sizes.

Here, we develop a theoretical framework that addresses these issues. Drawing on modern machine-learning theory [29–34], we propose a parameterization for RNNs trained with backpropagation through time that guarantees consistent population-level behavior in large networks and, crucially, allows us to titrate the degree of structure and disorder in the learned connectivity. The titration is controlled by a single parameter γ. As $\gamma \to 0^{+}$, recurrent weights remain unstructured and only the readout is learned, yielding a reservoir regime [35, 36]. As γ increases, recurrent connectivity is progressively restructured by learning, producing task-dependent internal representations. Varying γ thus generates a continuous family of task-compatible solutions whose internal dynamics differ in a controlled and interpretable way.

To characterize this family, we develop a dynamical mean-field theory (DMFT) describing the network in the limit of a large number of neurons. The DMFT shows that the population-averaged correlation function is governed by an action whose minimum determines the network’s macroscopic behavior. The action contains two competing terms, one inherited from the classical theory of chaotic random networks [18] that favors unstructured connectivity and high-dimensional activity, and another arising from learning that pulls the network toward task-relevant, low-dimensional representations. The parameter γ controls the relative strength of these terms, and the learned network reflects the outcome of the competition. A key prediction is that population-level statistics converge to a deterministic limit, while individual neurons remain independent samples from a single-neuron response distribution whose form is also set by γ. In the reservoir regime, this distribution is Gaussian, reflecting random connectivity. Recurrent restructuring drives it toward task-dependent, non-Gaussian forms. At the level of weights, this restructuring pulls outlier eigenvalues out of the random bulk, introducing dynamical modes that carry the learned temporal structure. For linear networks, increasing γ reshapes the effective temporal filter, amplifying task-relevant frequencies. For nonlinear networks, it drives a phase transition from chaotic, high-dimensional activity to ordered, low-dimensional dynamics that support generalization to time points beyond the training window.

We apply this framework to a motor task, previously studied by Sussillo et al. [27], in which an RNN must reproduce electromyographic (EMG) signals recorded from eight muscles during macaque reaching. By varying γ, we obtain a family of networks that all reproduce the EMG targets with comparable accuracy but differ systematically in their internal dynamics. Comparing these networks to simultaneous motor-cortex recordings, we find that reservoir networks $\left(\gamma \to 0^{+}\right)$ produce accurate muscle outputs but poorly match neural responses. An intermediate degree of recurrent restructuring substantially improves this match, though the strength of the improvement varies across metrics. The best-matching networks occupy a regime where learned dynamical structure coexists with random heterogeneity. More broadly, this picture suggests that large recurrent circuits may operate similarly, with varying degrees of structured recurrence embedded within otherwise random connectivity, sufficient to support generalizable, feature-based representations^2^.

## 2. Results

### 2.1 Recurrent-network model and training

We consider rate-based recurrent neural networks (RNNs) of N neurons. Each neuron i=1,…,N has a preactivation $x_{i}(t)$ and an activation $\phi_{i}(t)\equiv \phi \left(x_{i}(t)\right)$, where ϕ(⋅) is a pointwise nonlinearity. As is typical, we interpret $x_{i}(t)$ as a filtered input current to neuron i and $\phi_{i}(t)$ as its firing rate. Except when otherwise specified, we use ϕ(⋅)=tanh(⋅) throughout this work.

The neurons are coupled through a matrix of recurrent weights $J_{ij}$ and receive $D_{\text{in}}$-dimensional time-dependent inputs $I_{a}(t)$, where $a=1,\ldots ,D_{\text{in}}$, via input weights $U_{ia}$. The network dynamics follow

(1) $$\tau \frac{dx_{i}(t)}{dt}=-x_{i}(t)+\frac{g}{\sqrt{N}}\sum_{j=1}^{N}J_{ij}\phi_{j}(t)+\sum_{a=1}^{D_{\text{in}}}U_{ia}I_{a}(t),$$

where τ is a single-neuron time constant that, until Sec. 2.6, we set to unity. The factor $g/\sqrt{N}$ ensures that the typical size of the recurrent input to each neuron remains fixed as N grows, and the gain parameter g controls the strength of recurrent interactions [18]. The network produces $D_{\text{out}}$-dimensional outputs $y_{a}(t)$, where $a=1,\ldots ,D_{\text{out}}$, through a linear readout with weights $V_{ia}$,

(2) $$y_{a}(t)=\frac{1}{N\gamma }\sum_{i=1}^{N}V_{ia}\phi_{i}(t).$$

**Figure 1:**
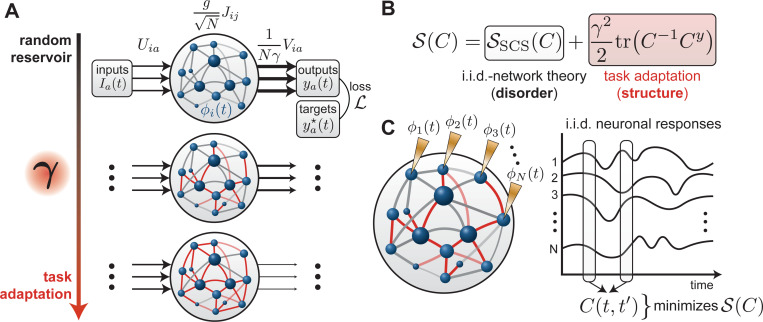
Framework overview. **(A)** Illustration of our task-trained RNN framework. An RNN receives time-dependent inputs, processes them through recurrent connectivity, and produces outputs through a linear readout. The network is trained to match target outputs by minimizing a loss. The parameter γ controls the degree to which learning reshapes recurrent connectivity, interpolating between a reservoir regime ($\gamma \to 0^{+}$, top) in which recurrent weights remain unstructured, and a task-adapted regime (large γ, bottom) in which learned structure progressively reshapes the recurrent connections. In machine learning theory, these are referred to as the lazy and rich regimes, respectively [29, 30]. **(B)** In the large-network limit, the population-averaged correlation function $C\left(t,t^{\prime }\right)$ is deterministic and minimizes an action 𝒮(C) composed of two competing terms. The first is inherited from the mean-field theory of random networks and favors chaotic, high-dimensional activity. The second arises from learning and drives the network toward temporal structure aligned with the task targets. **(C)** Individual neurons in the large-network limit are independent and identically distributed (i.i.d.) samples from a distribution over response profiles. That is, each neuron produces a temporal trace that is a random draw from a distribution, with the form of this distribution depending on the degree of recurrent restructuring.

The parameter γ>0 plays a central role in what follows, serving as our primary knob for titrating the degree of recurrent restructuring. A scaling argument explains why. The activations $\phi_{i}(t)$ and the readout weights $V_{ia}$ are both 𝒪(1) in N. When the activations have generic alignment with the columns of the readout weight matrix, as is the case at initialization and in the absence of recurrent restructuring, the sum $\sum_{i=1}^{N}V_{ia}\phi_{i}(t)$ grows as $\sqrt{N}$. After the $1/(\sqrt{N}\gamma )$ prefactor in the readout, the output therefore shrinks as $1/(\sqrt{N}\gamma )$, vanishing as N→∞ for any fixed γ. To produce a finite output, the activations $\phi_{i}(t)$ must develop alignment with the columns of $V_{ia}$, requiring the internal representation to adapt to the task. Larger γ demands stronger alignment and thus more restructuring of the recurrent weights. Smaller γ allows the $1/(\sqrt{N}\gamma )$ prefactor to compensate, so that even weak alignment suffices. In the limit $\gamma \to 0^{+}$, the recurrent weights are not adapted by learning at all, and the network operates as a reservoir [35, 36, 38], with only the readout weights effectively trained (Fig. 1A). In the machine-learning theory community, this phenomenon is known as *feature learning* [34], with the small-γ and large-γ limits referred to as the *lazy* and *rich* regimes, respectively [29]. The parameter scalings we have adopted correspond to the so-called *maximal-update parameterization* (*μ*P) [30–32, 34], which ensures that the degree of feature learning remains nontrivial and consistent as N grows.

We train the network in an end-to-end manner, meaning that the recurrent weights J, input weights U, and readout weights V, collected in the parameter set Θ, are all learned jointly. The training objective is to make the network output $y_{a}(t)$ match a target sequence $y_{a}^{⋆}(t)$ over a time interval [0,T]. We define a loss function, interpretable within our theoretical framework as an energy, consisting of a mean-squared-error (MSE) data term and a quadratic or ridge regularizer,

(3) $$E(\text{Θ})=\frac{N\gamma^{2}}{2D_{\text{out}}T}\int_{0}^{T}dt\sum_{a=1}^{D_{\text{out}}}{\left(y_{a}(t)-y_{a}^{⋆}(t)\right)}^{2}+\frac{1}{2\beta }\|\text{Θ}{\|}^{2},$$

where $\|\text{Θ}{\|}^{2}$ is the sum of squares of all parameters and the prefactor $N\gamma^{2}$ ensures compatibility between the data term and the output scaling in Eq. (2). The output $y_{a}(t)$ depends on the parameters Θ through the recurrent dynamics of Eqs. (1)–(2), making this a highly nonlinear optimization problem. The scalar β>0 controls both the strength of the ridge regularization and the amplitude of the learning noise introduced below. The factor of 1/β in the ridge term ensures that even in the limit β→∞, where the noise vanishes and the data-fit term dominates, the regularization also vanishes, so that the learning dynamics sample a degenerate manifold of solutions rather than converging to a single point [39]. The formulation extends straightforwardly to multiple input-output sequence pairs by averaging the data term over a set of sequences (see Sec. 2.6).

We learn the parameters through a continuous-time Langevin dynamics in which the parameters Θ evolve over a learning time s,

(4) $$\frac{d\text{Θ}(s)}{ds}=-\nabla_{\text{Θ}}E(\text{Θ}(s))+\sqrt{\frac{2}{\beta }}\xi (s),$$

where ξ(s) is white noise, independent across parameters and learning time. The first term is the gradient of the energy, computed via backpropagation through time, and drives the parameters toward configurations that fit the target while keeping weights small. The second term injects noise whose amplitude is controlled by β, so that larger β corresponds to less noise and a stronger emphasis on minimizing the energy. We refer to this as Langevin gradient flow. A key property is that as s→∞, the distribution over parameters converges to the Gibbs distribution, P(Θ)∝exp(−βE(Θ)). At large β, this distribution concentrates on low-energy configurations, that is, networks that fit the target well while keeping weights moderate. Working with the Gibbs distribution allows us to characterize the statistical properties of trained networks at equilibrium without tracking the full learning trajectory.

The Gibbs distribution has a natural Bayesian interpretation as a posterior that balances an independent and identically distributed (i.i.d.) Gaussian prior on the weights against a likelihood that rewards alignment between the network’s temporal correlations and those of the target. The prior favors random, unstructured connectivity; the likelihood favors task-relevant structure. The parameter γ controls how far the posterior departs from the prior, titrating the mixture of randomness and task structure in the learned connectivity [34, 39–41].

### 2.2 Large-network limit and dynamical mean-field theory

In the large-N limit, the network can be analyzed using dynamical mean-field theory (DMFT). The core idea is to reduce the N-dimensional dynamics to a small number of self-consistent equations for order parameters (summary statistics) that describe the population-level behavior. These order parameters are self-averaging, meaning that they can be measured in finite networks and agree with the DMFT predictions up to fluctuations that shrink as $1/\sqrt{N}$, independent of the particular weight realization [42]. DMFT therefore provides an exact description of the population-level dynamics as N→∞.

The central order parameter is the population-averaged temporal correlation function,

(5) $$C\left(t,t^{\prime }\right)=\frac{1}{N}\sum_{i=1}^{N}\phi_{i}(t)\phi_{i}\left(t^{\prime }\right),$$

which measures the similarity of the network’s internal representation between times t and $t^{\prime }$. For a random network with i.i.d. weights and no learning, the DMFT was developed by Sompolinsky, Crisanti, and Sommers (SCS) [18]. One way to formulate their theory, which will prove natural for incorporating learning, is in terms of an action ${𝒮}_{\text{SCS}}(C)$ that $C\left(t,t^{\prime }\right)$ minimizes. The action plays a role analogous to a loss function, but for the population-level structure of the network rather than its parameters; the physical correlation function is the one that minimizes it. The SCS action encodes the statistics of a single neuron driven by a self-consistent Gaussian process whose covariance depends on $C\left(t,t^{\prime }\right)$ itself (Sec. SI.1). Our theory shows that learning adds a second term to this action. In the β→∞ limit, the correlation function of the learned network minimizes

(6) $$𝒮(C)={𝒮}_{\text{SCS}}(C)+\frac{\gamma^{2}}{2}\mathrm{tr}\left(C^{-1}C^{y}\right),$$

where $C^{y}\left(t,t^{\prime }\right)=\sum_{a=1}^{D_{\text{out}}}y_{a}^{⋆}(t)y_{a}^{⋆}\left(t^{\prime }\right)$ is the temporal correlation of the target outputs, and the trace and inverse in the second term treat $C\left(t,t^{\prime }\right)$ and $C^{y}\left(t,t^{\prime }\right)$ as matrices with temporal indices. The structure of this action makes the competition between randomness and task structure explicit (Fig. 1B). The first term, inherited from the classical SCS theory, encodes the statistics of a random, unstructured network. The second term penalizes misalignment between the network’s temporal correlations and the target. When $C\left(t,t^{\prime }\right)$ has little power along temporal modes present in $C^{y}\left(t,t^{\prime }\right)$, the trace $\mathrm{tr}\left(C^{-1}C^{y}\right)$ is large, driving $C\left(t,t^{\prime }\right)$ to better capture those modes. The strength of this drive is set by $\gamma^{2}$, so as $\gamma \to 0^{+}$ the action reduces to ${𝒮}_{\text{SCS}}(C)$ and the network behaves as a reservoir, while at large γ the learned term dominates and the correlation structure is shaped heavily by the task. In the β→∞ limit, the network output matches the target exactly, $y_{a}(t)=y_{a}^{⋆}(t)$, provided $C\left(t,t^{\prime }\right)$ is invertible (Eq. (SI.13)). At finite β, the inverse $C^{-1}\left(t,t^{\prime }\right)$ in the action is replaced by a regularized inverse, and the output incurs a computable discrepancy from the target (Sec. SI.1).

### 2.3 Learning reshapes the single-neuron response distribution

At large N, while the correlation function $C\left(t,t^{\prime }\right)$ is deterministic, individual neurons are independent, identically distributed samples from a distribution over response profiles (Fig. 1C). Neurons are thus heterogeneous at all values of γ, but the character of this heterogeneity changes with γ, reflecting the mixture of randomness and task structure in the underlying connectivity. We now describe this distribution by unpacking the structure of the action.

The SCS action ${𝒮}_{\text{SCS}}(C)$ contains an inner optimization over a conjugate (or auxiliary) variable $\hat{C}\left(t,t^{\prime }\right)$, so that the full variational problem is a saddle point (Sec. SI.1). A saddle point is a stationary point of the action (where all gradients vanish) that is a minimum in some directions and a maximum in others; here, the action is minimized over $C\left(t,t^{\prime }\right)$ and maximized over $\hat{C}\left(t,t^{\prime }\right)$. The stationarity condition with respect to $\hat{C}\left(t,t^{\prime }\right)$ produces a self-consistency equation that determines $C\left(t,t^{\prime }\right)$, while the saddle-point value of $\hat{C}\left(t,t^{\prime }\right)$ itself controls how the distribution over single-neuron responses departs from a baseline Gaussian. In the SCS DMFT, each neuron receives an input current η(t) drawn independently from a Gaussian process with mean zero and covariance $\langle \eta (t)\eta \left(t^{\prime }\right)\rangle =g^{2}C\left(t,t^{\prime }\right)+C^{I}\left(t,t^{\prime }\right)$, where $C^{I}\left(t,t^{\prime }\right)$ is the covariance of the external inputs. This input current replaces the actual recurrent input from other neurons, capturing its statistics in a self-consistent way. The current is passed through the single-neuron dynamics $\left(1+\partial_{t}\right)x(t)=\eta (t)$ to produce the preactivation x(t), which, because this is a linear filter of a Gaussian process, is also Gaussian. Applying the nonlinearity then gives the activation ϕ(t)=ϕ(x(t)). In the learned system, a crucial step is inserted into this process, namely, the Gaussian distribution over input currents is reweighted by a tilting factor $\propto \exp\left(\frac{1}{2}\int_{0}^{T}dt\int_{0}^{T}dt^{\prime }\hat{C}\left(t,t^{\prime }\right)\phi (t)\phi \left(t^{\prime }\right)\right)$, where ϕ(t) is the activation produced by η(t) through the single-neuron dynamics. This tilting biases the population toward neurons whose input currents produce activations aligned with the structure encoded in $\hat{C}\left(t,t^{\prime }\right)$. Self-consistency requires that the correlation function computed under this tilted distribution matches the order parameter $C\left(t,t^{\prime }\right)$ that enters the action.

As $\gamma \to 0^{+}$, the learning term in the action (6) vanishes and the saddle-point value of the conjugate variable is $\hat{C}\left(t,t^{\prime }\right)=0$. The tilting factor reduces to a constant, the input current η(t) remains a Gaussian process, and the SCS theory is recovered. When γ>0, the learning term shifts the saddle-point value of $C\left(t,t^{\prime }\right)$ away from the random-network solution, which in turn drives $\hat{C}\left(t,t^{\prime }\right)$ away from zero. The resulting tilting makes the distribution over input currents non-Gaussian, driving the preactivation and activation distributions away from Gaussianity in manner that depends on the task through $C^{y}\left(t,t^{\prime }\right)$. Thus, although neurons are always i.i.d. samples from a population distribution, the distribution itself is progressively reshaped by learning. As $\gamma \to 0^{+}$ it is Gaussian, reflecting purely random connectivity, while at larger γ it acquires non-Gaussian, task-relevant structure. Intuitively, recurrent restructuring enriches the population with neurons whose temporal responses are useful for the task, at the expense of those that are not. The tilting factor implements this enrichment, exponentially upweighting input current realizations whose resulting activations have two-point structure aligned with $\hat{C}\left(t,t^{\prime }\right)$, which the saddle-point conditions tie to the task target $C^{y}\left(t,t^{\prime }\right)$. Non-Gaussianity in single-neuron statistics is the imprint of this task-driven enrichment.

### 2.4 Recurrent restructuring amplifies task-relevant frequencies in linear networks

As $\gamma \to 0^{+}$ (the SCS theory), the distribution over single-neuron responses is Gaussian, and the self-consistency equations for the correlation function can be solved analytically. At γ>0, the non-Gaussian tilting introduced by learning necessitates numerical solutions (Sec. SI.1). To build analytical intuition for how recurrent restructuring reshapes activity, we consider the one case in which a closed-form solution can still be obtained, namely linear recurrent networks, with ϕ(⋅) the identity. Linear networks are limited as models of biological circuits and cannot exhibit phenomena such as chaos, but afford a tractable testbed. The dynamics are linear, but the learning process that produces the trained weights is not [34, 43, 44], so obtaining exact results for the learned representations is nontrivial.

A further simplification arises if we assume that the inputs, outputs, and network activity are statistically stationary, meaning that correlation functions depend only on time differences. Under this assumption, the DMFT equation for the power spectrum C(ω) decouples across frequencies, reducing to a quadratic equation at each ω in the β→∞ limit (Sec. SI.3.3). This yields a closed-form expression for the power spectrum of the learned network in terms of the input and target power spectra,

(7) $$C(\omega )=\frac{1+2\gamma^{2}g^{2}\frac{C^{y}(\omega )}{C^{I}(\omega )}+\sqrt{1+4\gamma^{2}\left(1+\omega^{2}\right)\frac{C^{y}(\omega )}{C^{I}(\omega )}}}{2\left(1+\omega^{2}-g^{2}-\gamma^{2}g^{4}\frac{C^{y}(\omega )}{C^{I}(\omega )}\right)}C^{I}(\omega ),$$

where $C^{I}(\omega )$ and $C^{y}(\omega )$ are the power spectra of the input and target, respectively. The ratio $C^{y}(\omega )/C^{I}(\omega )$ measures how much each frequency is over-represented in the target relative to the input, and learning reshapes the network’s transfer function to amplify precisely those frequencies.

**Figure 2:**
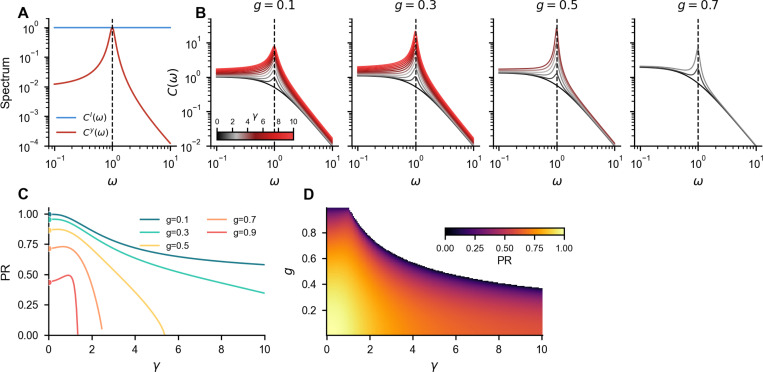
Recurrent restructuring amplifies task-relevant frequencies in linear networks. **(A)** Linear networks are trained to transform white-noise input with flat power spectrum $C^{I}(\omega )=1$ into outputs whose power spectrum is concentrated around a preferred frequency $\omega_{⋆}=1$ (dashed vertical line). **(B)** Power spectra C(ω) of the learned network activity for different values of the gain g (panels). Within each panel, increasing γ (indicated by hue) progressively amplifies power near the target frequency. For a given g, there is a maximum γ beyond which the linear dynamics become unstable (Sec. SI.3.3); only stable parameter combinations are shown. **(C)** Participation ratio of the stationary covariance (defined in main text) as a function of γ for several values of g. **(D)** PR over the (γ,g) plane. The blank region corresponds to unstable linear dynamics.

We first examine the reservoir limit $\left(\gamma \to 0^{+}\right)$, in which Eq. (7) reduces to $C(\omega )=C^{I}(\omega )/\left(1+\omega^{2}-\right.\left.g^{2}\right)$. For white-noise input ($C^{I}(\omega )=1$; Fig. 2A), this is the power spectrum of an Ornstein-Uhlenbeck process with correlation time $1/\sqrt{1-g^{2}}$, which diverges as $g\to 1^{-}$, reflecting the approach to instability of the random linear network. The reservoir power spectrum decays monotonically from low to high frequencies (Fig. 2B, $\gamma \to 0^{+}$ curves in each panel).

We now consider the effect of increasing γ. We illustrate with a network trained to transform white-noise input into noise whose power spectrum is sharply peaked around a preferred frequency $\omega_{⋆}=1$, with target spectrum $C^{y}(\omega )=1/\left(1+10^{2}{\left(|\omega |-\omega_{⋆}\right)}^{2}\right)$ (Fig. 2A). As γ increases from zero, a peak emerges around the target frequency and grows progressively (Fig. 2B). Recurrent restructuring thus selectively amplifies the frequencies demanded by the task. Equation (7) also implies a stability condition, namely that the denominator must remain positive for all ω, which places an upper bound on γ for a given g and target spectrum. For each value of g, we show power spectra only for values of γ at which the linear dynamics remain stable.

We summarize the effect of recurrent restructuring on the population-level dynamics using the participation ratio (PR) of the power spectrum, $\text{PR}=C{\left(0\right)}^{2}/\int_{-\infty }^{\infty }d\tau C{\left(\tau \right)}^{2}$, which measures the number of dimensions explored by the network per unit of time [45]. In the reservoir limit, $\text{PR}=\sqrt{1-g^{2}}$, which vanishes as $g\to 1^{-}$, consistent with the diverging correlation time noted above [46]. With increasing γ, the participation ratio first increases, prominently so for large g, and then decreases toward zero as the linear dynamics approach the stability boundary (Fig. 2C,D). This nonmonotonic dependence of dimensionality on γ will reappear in the nonlinear networks studied below.

A linear network can only output frequencies already present in its input and cannot autonomously generate temporal structure. We turn next to nonlinear networks, where richer dynamical phenomena become possible.

### 2.5 Recurrent restructuring suppresses chaos and enables temporal generalization

A hallmark of task-adapted representations in feedforward networks is that they support generalization [30, 47–52]. We now ask whether an analogous phenomenon occurs in the temporal domain, that is, whether restructuring recurrent connectivity enables generalization across time. Unlike the linear networks analyzed above, nonlinear networks can generate novel temporal structure autonomously, and we therefore turn to a common task in the RNN literature that probes exactly this question, the autonomous generation of a periodic signal [28, 53]. Producing a stable oscillation requires the suppression of chaotic dynamics, making this task a natural setting for understanding how recurrent restructuring reshapes the dynamical regime of the network.

**Figure 3:**
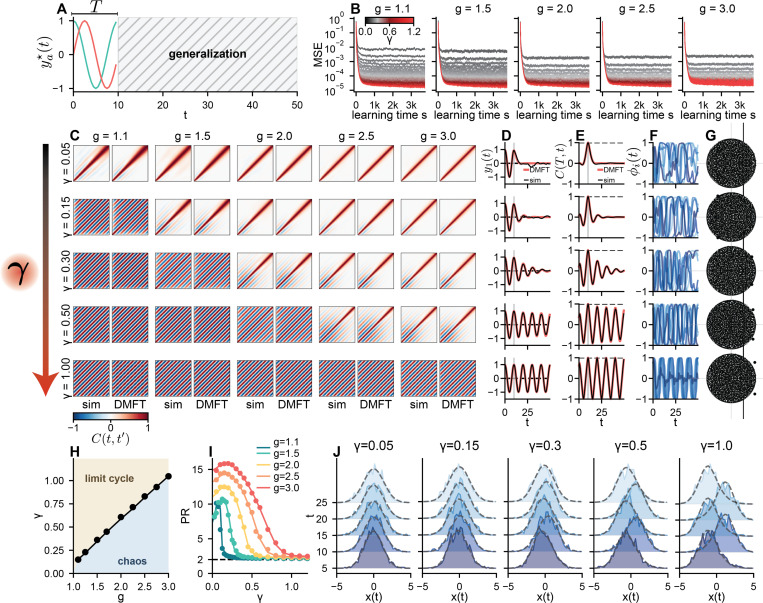
Recurrent restructuring suppresses chaos and enables temporal generalization. **(A)** Target signal $y_{a}^{⋆}(t)$: a single period (T=10) of a two-dimensional sinusoidal output. We asked what dynamics trained networks produced at later times, $T<t\leq T_{\text{tot}}$, where $T_{\text{tot}}=50$ (generalization window). **(B)** For each pair (g,γ), we trained 10 independent networks of size N=2500 via Langevin gradient flow (β=2000). Here we show learning curves for different values of γ (lines) and g (columns). All networks achieved low training error, with smaller residuals at larger γ (a finite-β effect; see main text). **(C)** Temporal correlation $C\left(t,t^{\prime }\right)$, over the $T_{\text{tot}}\times T_{\text{tot}}$ window, for different values of γ (rows) and g (columns), comparing simulation (left in each panel, averaged across 10 networks) and DMFT (right in each panel). **(D)** Network output $y_{1}(t)$ across the training and test periods, for g=2 and the same γ values as in (C). Solid lines show the output of a single trained network; dashed lines show the DMFT prediction obtained by kernel regression from the training-window correlation function (Eq. (SI.33)). **(E)** Slices C(T,t) of the temporal correlation for g=2 and the same γ values as in (C), comparing simulation (thin lines) and DMFT (thick lines). Vertical line indicates t=T. **(F)** Single-neuron activation traces across the training and test periods for g=2 and the same γ values as in (C). **(G)** Eigenvalue spectra of $\frac{g}{\sqrt{N}}J$ for g=2 and the same γ values as in (C). Black dots show eigenvalues from a single network, and gray dots show eigenvalues from the remaining 9 networks. At small γ, eigenvalues follow the circular law with radius g. Increasing γ pulls complex-conjugate outlier pairs out of the bulk. The vertical line indicates the stability boundary, Re (λ)=1. **(H)** Phase diagram in the (g,γ) plane, with the curve $\gamma^{⋆}(g)$ separating the chaotic regime (large g, small γ) from the limit-cycle regime (small g, large γ); shaded regions are labeled accordingly. Dots show simulation, line shows DMFT. **(I)** Participation ratio of $C\left(t,t^{\prime }\right)$ over the training and test periods as a function of γ for different values of g, comparing simulation (dots, computed from the mean correlation across 10 networks) and DMFT (lines). **(J)** Preactivation distributions at selected time points for g=2 and the same γ values as in (C), comparing simulation (filled histograms, 100 values collected from each of the 10 networks per γ) and DMFT (dashed outlines, includes tilting).

We trained networks to produce a single period of a two-dimensional sinusoidal output with period *T* = 10 time constants, with no external input (Fig. 3A). The target returns to its initial value at time T, which is compatible with periodicity but does not imply it, since the network sees only one period and has no opportunity to learn from repetitions. After training, we ran the network beyond the training window and examined its generalization behavior. We restrict to the regime g>1, in which the dynamics without recurrent restructuring are chaotic, as is standard in practice when training RNNs [53].

We trained networks and solved the DMFT equations numerically across a grid of (g,γ) values (Sec. SI.2, SI.5). All configurations achieve low training error (Fig. 3B), with smaller errors at larger γ. Any nonzero error is a finite-β effect, since the β→∞ readout reproduces the target exactly whenever $C\left(t,t^{\prime }\right)$ is invertible (Eq. (SI.13)). To leading order in 1/β, the residual is proportional to $C^{-1}\left(t,t^{\prime }\right)Y^{⋆}$, with squared norm ${\left(Y^{⋆}\right)}^{⊤}C^{-2}\left(t,t^{\prime }\right)Y^{⋆}$. The action’s learning term penalizes ${\left(Y^{⋆}\right)}^{⊤}C^{-1}\left(t,t^{\prime }\right)Y^{⋆}$ with prefactor $\gamma^{2}$, the same quadratic form in $Y^{⋆}$ but with $C^{-1}$ in place of $C^{-2}$. Larger γ shrinks both, and the residual along with them.

Despite comparable training performance, networks at different γ differ markedly in their internal dynamics and generalization behavior. Fig. 3C displays the correlation function $C\left(t,t^{\prime }\right)$ for each (g,γ) configuration, computed both from finite-N simulations and from the DMFT. The DMFT provides an extremely close match throughout, as confirmed by the overlaid slices C(T,t) in Fig. 3E. Beyond population-level statistics, the DMFT also predicts the network’s autonomous output in the test window via a kernel regression on the training-window correlation function (Eq. (SI.33), Sec. SI.2.2), and the prediction matches simulation closely throughout (Fig. 3D). At small γ, $C\left(t,t^{\prime }\right)$ decays rapidly away from the diagonal, the signature of chaotic dynamics with short-lived temporal correlations. As γ increases, oscillatory structure emerges and grows until, at large γ, it dominates and the chaotic decay is no longer visible.

Single-neuron activity traces reflect this same progression (Fig. 3F). Recall that individual neurons are i.i.d. samples from a distribution that is progressively reshaped by recurrent restructuring. At small γ, each neuron’s response is a smooth but random temporal fluctuation, and the population looks disordered. At large γ, responses converge toward sinusoids passed through the nonlinearity ϕ(⋅), differing only in phase, and the appearance of disorder is lost.

This progression culminates in a transition between chaotic and ordered dynamics. Following [54], we identified the onset of nonchaotic, limit-cycle dynamics by checking whether the normalized correlation function returned to unity after a time lag, indicating that the population vector of activity returns exactly to itself. We assessed this after 3T of autonomous running to allow convergence to a putative limit cycle. This criterion defines a critical value $\gamma^{⋆}(g)$ above which the dynamics are nonchaotic, partitioning the (g,γ) plane into chaotic and limit-cycle regimes (Fig. 3H). The DMFT predicts this phase boundary accurately. Larger g requires larger γ to suppress chaos, as stronger recurrent gain produces more vigorous chaotic fluctuations that recurrent restructuring must overcome.

In the non-stationary setting considered here, we compute a participation ratio over a finite observation window, $\text{PR}={\left(\int_{0}^{T_{\text{tot}}}dtC(t,t)\right)}^{2}/\int_{0}^{T_{\text{tot}}}dt\int_{0}^{T_{\text{tot}}}dt^{\prime }C^{2}\left(t,t^{\prime }\right)$, which counts the number of dimensions explored by network activity over this window, where $T_{\text{tot}}=50$ (Fig. 3I). Unlike the stationary PR of Sec. 2.4, which measures a rate, this is a dimensionless count; the two correspond to different orders of limits in N and the observation time $T_{\text{tot}}$ (Sec. SI.4). As γ increases from zero, PR first rises, then falls, eventually approaching two, the value expected for a pure sinusoidal oscillation. This nonmonotonicity arises because at small γ all variance comes from high-dimensional chaos, at large γ it comes from the two-dimensional oscillation, and at intermediate γ both sources contribute. This is qualitatively reminiscent of the nonmonotonic participation ratio observed in the linear case (Fig. 2C), despite the different definitions.

Fig. 3J shows the distributions of preactivations across RNN neurons at selected time points, comparing simulations with the DMFT. At small γ, distributions are Gaussian and centered at zero at all times, as expected from the SCS theory. As γ increases, the distributions develop mild skewness, and the peak oscillates back and forth over time rather than remaining centered at zero, both of which reflect the tilting mechanism of the DMFT.

The quantities examined so far (namely, the correlation function, neuronal traces, and preactivation distributions) are all accessible through the DMFT. To understand the recurrent restructuring underlying these changes at the level of the weights, we examined the eigenvalue spectra of the recurrent weight matrix $\frac{g}{\sqrt{N}}J$ (Fig. 3G). As $\gamma \to 0^{+}$, eigenvalues are uniformly distributed within a disk of radius g, following the circular law for i.i.d. random matrices. Increasing γ pulls a pair of complex-conjugate outlier eigenvalues out of the bulk, with their distance from the bulk growing with γ, corresponding to progressively stronger oscillatory dynamics. This spectral structure provides a complementary view of the competition between randomness and task structure that the DMFT formalizes. In particular, the random bulk reflects the disordered component of single-neuron responses, while the outlier eigenvalues carry the learned oscillatory dynamics required for the task.

#### Frequency amplification, nonlinearly revisited

With the nonlinear DMFT in hand, we can revisit the frequency-amplification analysis of Sec. 2.4, now asking a *nonlinear* network, in the time-translation-invariant setting, to *autonomously* generate a target whose power is concentrated at frequency $\omega_{⋆}$ (rather than asking a linear network to produce such a target in response to a white-noise input; Sec.SI.3.4). We take the target to be a pure sinusoid. This target would be problematic for a linear network, with singularities produced in the transfer function (Eq. (7)). The nonlinearity resolves this because application of ϕ(⋅) in the time domain globally reshapes the power spectrum, producing continuous spectral content (with peaks at odd harmonics of $\omega_{⋆}$; Fig. SI.2). The nonlinearity also enables a qualitatively new phenomenon. In a linear network, frequencies are not mixed; the activity at any given frequency depends only on the input at that same frequency. Learning therefore has only one job, namely to amplify the frequencies that appear in the target, as seen in the power spectra of Fig. 2B. There is nothing to suppress, because the linear activation cannot generate power at frequencies absent from the input. The nonlinearity changes this. By acting pointwise in time, ϕ(⋅) produces harmonics and otherwise spreads power across frequencies, so the learning term must do double duty, both amplifying task-relevant frequencies and *suppressing* task-irrelevant ones. The DMFT solution makes this manifest. The conjugate variable $\hat{C}(\omega )$, which in the linear theory is non-negative everywhere (Eq. (SI.49)), now becomes positive near $\omega_{⋆}$, where it amplifies, and broadly negative elsewhere, where it suppresses (Fig. SI.2). The harmonics produced by the nonlinearity itself fall within this suppressed region and are pruned along with the rest of the task-irrelevant spectral content.

### 2.6 A family of networks that solve a macaque reaching task

The preceding sections illustrated our framework in simplified settings, with frequency amplification in linear networks and suppression of chaos with temporal generalization in nonlinear networks. In both cases, recurrent restructuring introduced task-relevant structure into an otherwise random network, pulling outlier eigenvalues out of the random spectral bulk, reshaping population-level-activity, and driving single-neuron statistics away from Gaussianity. We now apply this framework to a realistic motor task and show that these same phenomena arise. In the next section, we ask which degree of recurrent restructuring best accounts for neural data.

**Figure 4:**
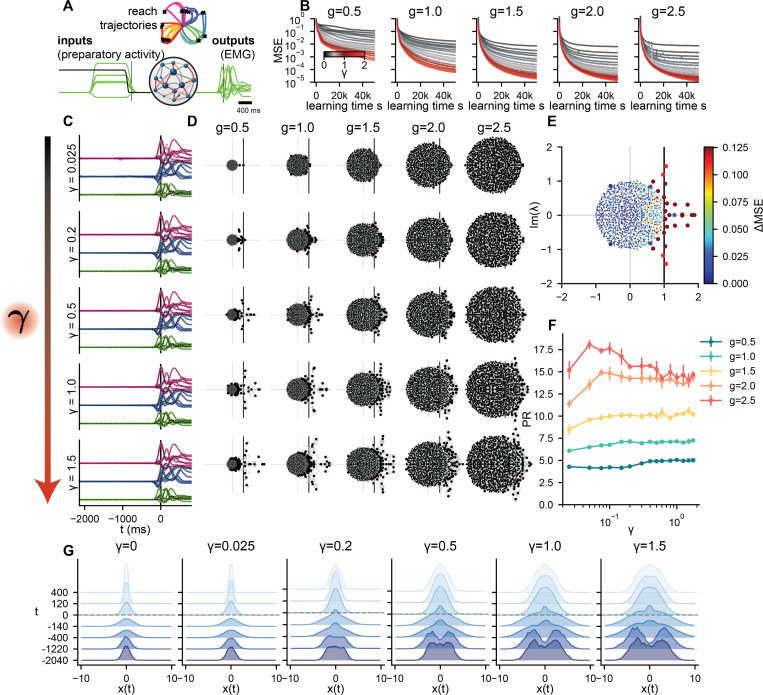
A family of networks that solve a macaque reaching task. **(A)** Task schematic. The network receives a six-dimensional condition-specific input derived from preparatory neural activity and a condition-independent hold cue (left), and produces eight-muscle EMG output (right). Inset: hand trajectories for all 27 reach conditions. **(B)** For each pair (g,γ), we trained 5 independent networks of size N=1024 via Langevin gradient flow $\left(\beta =10^{6}\right)$. Here we show the MSE as a function of learning time s for different values of γ (lines) and g (columns), averaged across 5 networks per condition. **(C)** EMG traces for g=1.5 and various values of γ (rows), showing three representative reach conditions (colors as in (A) inset). Translucent colored lines show the trained RNN output; thin dashed black lines show the target (measured) EMG. The vertical line indicates movement onset (t=0). **(D)** Eigenvalue spectra of $\frac{g}{\sqrt{N}}J$ for the same γ values as in (C) (rows) and different values of g (columns). Black dots show eigenvalues from a single network, and gray dots show eigenvalues from the remaining networks. The vertical line indicates the stability boundary, Re (λ)=1. **(E)** Eigenvalue spectrum for g=1 and γ=0.3, with each eigenvalue colored by the increase in MSE (ΔMSE) when the corresponding mode was ablated. **(F)** Participation ratio of RNN activity computed over a ±400 ms window around movement onset, as a function of γ for different values of g (colors). **(G)** Preactivation distributions at selected time points for g=1 and the same γ values as in (C), plus an untrained network $\left(\gamma \to 0^{+}\right)$.

The task is drawn from Churchland et al. [6], in which a macaque performed 27 reaching movements, including both direct reaches and curved reaches around obstacles, producing diverse hand trajectories (Fig. 4A, inset) and muscle activation patterns. Following Sussillo et al. [27], who originally studied this task in the task-trained RNN framework, we trained an RNN to transform temporally simple inputs into the temporally complex EMG patterns recorded from 8 muscles during these reaches. The network receives two types of input (Fig. 4A, left). The first is a six-dimensional condition-specific signal derived from the top principal components of the recorded preparatory neural activity, which specifies which of the 27 reaches to perform. The second is a condition-independent hold cue whose offset precedes movement. We study monkey J, as analyzed in the main text of Sussillo et al. [27]; simultaneous neural recordings from M1 and PMd are from Churchland et al. [6] and will be used for comparison in the next section. We set the time constant to τ=50ms and trained networks of N=1024 neurons across a grid of g and γ values, with 5 independent runs per configuration via Langevin gradient flow (Sec. SI.2). Task parameters were matched to Sussillo et al. [27] wherever possible (Table SI.2; Sec. SI.6). For this task, the DMFT equations cannot currently be solved numerically because of issues of computational complexity and stability, an important avenue for future work. The theory nonetheless guarantees existence of a large-N limit, provides expectations for the role of γ, and identifies which summary statistics are constrained by the task [55].

All configurations produce accurate EMG output (Fig. 4C), with the MSE low throughout the (g,γ) grid (Fig. 4B), though somewhat higher at small g and small γ. Even near the reservoir limit, the network closely tracks the target muscle activity across conditions. By varying γ, we thus obtain a family of networks that all solve the same task but differ systematically in their internal dynamics, providing a controlled way to navigate the space of task-compatible solutions. Sussillo et al. [27] explored a version of this idea by comparing two networks trained with different regularization schemes, one that matched neural data well and one that did not, but without the ability to continuously modulate the nature of recurrent restructuring, and without a theory linking such restructuring to internal dynamics. Our framework places this comparison on systematic footing, generating a continuous family whose internal representations vary in an interpretable way.

We examined the eigenvalue spectra of the recurrent weight matrix (Fig. 4D). As in the sine wave task (Fig. 3G), outlier eigenvalues emerge from the random bulk with increasing γ, but here the spectral structure is richer, reflecting the higher-dimensional nature of the task. Several outliers occur in complex-conjugate pairs with large imaginary parts, corresponding to oscillatory modes consistent with the quasi-oscillatory dynamics identified in motor cortex by Churchland et al. [6]. Sussillo et al. [27] observed similar spectral structure by linearizing their regularized model around its fixed point. We confirmed the functional relevance of these outliers by ablating individual eigenvalues and measuring the resulting change in MSE (Fig. 4E). Removing outlier eigenvalues, particularly those with large imaginary parts, substantially degrades task performance, while removing bulk eigenvalues has negligible effect.

We computed the participation ratio of $C\left(t,t^{\prime }\right)$ over a ±400 ms window around movement onset as a measure of effective dimensionality (Fig. 4F). As in the linear (Fig. 2C) and sine wave (Fig. 3I) cases, PR is nonmonotonic for large g, first rising as task-relevant modes are added to the high-dimensional chaotic activity, then falling as those modes come to dominate. This nonmonotonicity is prominent at large g because it is in this regime that the inputs fail to suppress chaotic activity, at least at small γ, so that both chaotic and task-relevant variance coexist.

Fig. 4G shows the distributions of preactivations across RNN neurons at representative time points.

As $\gamma \to 0^{+}$, preactivations are approximately Gaussian, as expected from random connectivity, with slight positive excess kurtosis reflecting temporal structure in the inputs. Increasing γ reshapes these distributions into non-Gaussian forms whose character depends on the phase of the task. Early in the trial, the distribution is bimodal. Around movement onset, it develops a sharp central peak. Later in the movement, it returns to a roughly Gaussian shape. These time-varying departures from Gaussianity are a manifestation of the tilting mechanism of Sec. 2.3.

The phenomena identified in simplified settings, namely outlier eigenvalues emerging from the random bulk, non-Gaussian single-neuron statistics, and nonmonotonic dimensionality, all carry over to this realistic motor task. We now ask which member of this family of task-compatible solutions best matches neural data.

### 2.7 Matching neural data requires an intermediate degree of recurrent restructuring

We have shown that varying γ produces a family of networks that all generate accurate muscle outputs but differ in their internal dynamics. We now ask whether any of these networks resemble the neural circuits that actually produce reaching movements.

**Figure 5:**
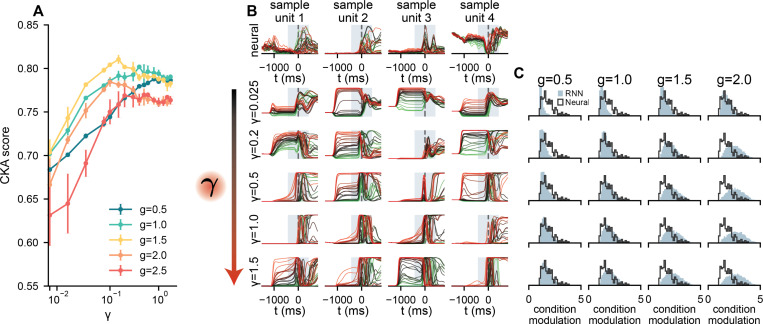
Matching neural data requires an intermediate degree of recurrent restructuring. **(A)** CKA score between RNN and M1/PMd population-level activity as a function of γ for different values of g (colors), computed in a ±400 ms window around movement onset. Error bars show standard deviation across runs. **(B)** Example single-neuron responses from M1/PMd recordings (top row) and RNN neurons at g=1 for various values of γ (rows below), with four sample neurons per row. Traces show all 27 reach conditions, colored green to red by preparatory activity. The shaded band indicates the ±400 ms movement window. **(C)** Distributions of per-neuron condition modulation (participation ratio of each neuron’s condition-by-time response matrix) for the same γ values as in (B) and different values of g (columns), comparing RNN (filled histograms, pooled across 5 runs) and M1/PMd recordings (black outlines).

We compared RNN population-level activity to simultaneously recorded M1/PMd neural data in a ±400 ms window around movement onset. We used centered kernel alignment (CKA) to measure similarity between the population-level correlation structure of the RNN and that of the neural recordings (Fig. 5A) [28, 56]. CKA is a natural metric in the context of our theory, since the correlation function is precisely the population-level quantity that the DMFT determines. Singular vector canonical correlation analysis, the metric used by Sussillo et al. [27], yields a similar picture, as does omitting the z-scoring of RNN and M1/PMd activities applied in Fig. 5A (Sec. SI.6, Fig. SI.5).

Similarity to neural data increases sharply with γ at small values, confirming that reservoir networks poorly match the structure of motor-cortex responses. For most parameter configurations with g>1, the curves peak at γ of order 0.1 and decline at larger values. This nonmonotonicity is more pronounced for some similarity metrics than others (Sec. SI.6). The robust finding is that an intermediate degree of recurrent restructuring substantially improves the match to neural data relative to the reservoir limit, while the largest values of γ do not improve it further and can worsen it.

We next examined the single-neuron correlates of this population-level alignment. Our theory predicts that individual neurons are i.i.d. samples from a distribution over response profiles whose character is controlled by γ, and that recurrent restructuring drives this distribution away from Gaussianity in a task-dependent manner. Fig. 5B displays example single-neuron responses from M1/PMd recordings alongside those from RNNs at several values of γ for g=1. At small γ, RNN neurons show little differentiation across reach conditions, with similar temporal profiles regardless of which reach is performed, reflecting the weak condition-based modulation provided by a marginally stable reservoir. As γ increases, neurons develop richer condition-dependent structure, including differentiated ramping activity during the delay period and diverse temporal profiles across conditions, increasingly resembling the responses of the M1/PMd neurons.

We quantified this by computing, for each neuron, the participation ratio of the singular values of its condition-by-time response matrix (this per-neuron measure is distinct from the population PR defined above). High values indicate condition-dependent modulation; low values indicate approximately condition-invariant activity. The distributions of this quantity across both M1/PMd and RNN neurons are shown in Fig. 5C. For RNNs, at small γ, and particularly for g≤1, these distributions are concentrated at low values, reflecting weak condition modulation. As γ increases, the distributions broaden and shift rightward, more closely resembling the distribution observed in M1/PMd recordings. At the largest values of γ, the distributions overshoot the neural reference for g>1 more substantially than they undershot it at small γ, paralleling the decline in population-level similarity seen for g>1 in Fig. 5A. The same intermediate regime of recurrent restructuring that best matches neural activity at the population level also best reproduces the statistics of individual neurons.

## 3 Discussion

We have developed a theoretical framework for task-trained RNNs in which a single parameter, γ, controls the degree to which learning reshapes recurrent connectivity. Our DMFT characterizes both the population-level and single-neuron consequences of this restructuring, showing that population-level quantities converge to a deterministic limit while individual neurons are i.i.d. samples from a (generally non-Gaussian) distribution whose character reflects the balance of randomness and structure in the underlying connectivity. Applied to simplified tasks, the theory yields analytical and numerical predictions for how recurrent restructuring amplifies task-relevant frequencies, suppresses chaos, and enables temporal generalization. Applied to a neuroscientifically motivated motor task, the framework generates a continuous family of networks that all produce accurate muscle outputs but differ in their internal dynamics, and we find that matching motor-cortex recordings requires an intermediate degree of recurrent restructuring.

These results bear on a basic question about neural circuits, namely how much learned structure they contain relative to disorder. The pervasive heterogeneity of single-neuron responses across many brain areas is consistent with a large degree of randomness in connectivity, yet the structured, low-dimensional population-level dynamics observed in motor cortex and elsewhere require some task-relevant recurrent structure. Our motor-cortex comparison is consistent with a picture in which this circuit is largely random but contains a small degree of learned recurrent structure sufficient to support the required dynamics. Whether this picture extends to circuits underlying cognitive tasks, where compositional and generalizable representations are essential [21, 22], is an important open question that our framework is well-positioned to address.

A recurring challenge when using task-trained RNNs as models of neural circuits is that each trained network yields a single internal solution, and it is unclear whether its resemblance to neural data reflects the structure of the task or incidental features of the optimization. To make progress, one needs a method for generating multiple task-compatible solutions that satisfies two desiderata. First, the source of diversity should be a controllable parameter with reproducible effects. Second, that parameter should have an interpretable impact on the learned representations, so that when a particular solution matches neural data, one can draw specific conclusions about what properties of the network were responsible.

Existing approaches fall short on one or both counts. Ad hoc regularization, of the type used in [27], provides some control over the learned solution, but without a theory linking regularization to its effects on internal dynamics, interpretation remains difficult [28]. Qian and Pehlevan [57] proposed a more systematic method in which previously found solutions are iteratively projected out to encourage the discovery of qualitatively distinct networks. This explores the solution space more thoroughly, but at considerable computational cost, and the structure of each successive solution is defined only by its dissimilarity from prior ones rather than by an interpretable parameter. Perhaps the most common approach is to train small networks with different random seeds, exploiting finite-size fluctuations to produce diverse solutions [28, 58–60]. However, such finite-size diversity is neither controllable nor interpretable.

Our framework satisfies both desiderata. The parameter γ continuously and controllably varies the degree of recurrent restructuring, and the DMFT provides an explicit theory of how it reshapes temporal correlations and single-neuron response distributions. When a particular value of γ best matches neural data, this directly quantifies the degree to which task learning has restructured recurrent connectivity in the circuit being modeled (as is evident, for example, when examining the eigenvalues of the learned connectivity; Fig. 4D,E). Importantly, this family of solutions persists in the large-N limit, providing a mechanism for diversity that does not rely on finite-size effects. We speculate that finite-size effects are unlikely to be a meaningful generator of diversity in real mammalian circuits. The human cortex contains roughly 10^10^ neurons, and while the more relevant quantity for scaling is the number of inputs per neuron (roughly 10^4^, which equals N in our fully connected networks), this is still large. If finite-size fluctuations were the relevant source of variability, brains this large should self-average toward a single prototypical solution. That individuals differ enormously in their behavioral strategies and cognitive styles suggests more structured mechanisms are at work, of which differences in the degree of recurrent restructuring provide one concrete example.

In related work, several papers considered feedforward networks in the lazy and rich regimes to understand the learned neural representations and algorithms underlying phenomena like context-dependent decision making [61], multitask cognition [62], equality reasoning [63], and the influence of the rank of the initial connectivity on learned representations [64]. For RNNs, Huang et al. [60] empirically studied solution degeneracy by varying network size, task complexity, regularization, and feature-learning strength (also using a *μ*P parameterization with parameter γ), and mapped how each factor shapes the space of solutions. Our theory focuses on the high rank regime and provides a precise account of what converges and what does not in the large-N limit. Population-level quantities such as the correlation function become deterministic, but a large degenerate manifold of weight-space solutions remains, across which individual weights and single-neuron responses vary. Exploring the structure of this manifold, as done by Huang et al. [60], is an interesting avenue for future work; we return to a related point when discussing representational drift below.

Schuessler et al. [65] observed that varying the magnitude of the readout weights, which corresponds to varying γ, leads to a transition between “lazy” and “rich” learning regimes, that is, a reservoir regime and a regime with recurrent restructuring. Focusing on the extremes, they identified the rich regime with an aligned readout in which the readout weights lie in the subspace spanned by the top principal components of activity, and the lazy regime with an oblique readout where they do not. They provided a mean-field account of these regimes for noise-driven linear networks in a stationary state, building on earlier work considering tasks depending on the fixed point of a linear RNN [66]. Some of the present authors subsequently analyzed the gradient flow learning dynamics of linear, non-stationary, noise-driven RNNs [67], focusing on the contrast between outlier eigenvalue emergence in an ultra-rich regime and vanishing dynamical change in a very lazy regime. In addition to being limited to linear systems, none of these works showed how solutions vary at intermediate values of γ, nor did they consider general sequence-to-sequence tasks. Our results reveal a wide array of behaviors in the (g,γ) plane, where larger g enhances the chaotic activity of the reservoir while γ counteracts it by introducing learned structure.

Our framework connects to, but differs from, the well-studied class of low-rank RNN models [68]. In our energy E(Θ), the Frobenius norm $\|J{\|}_{F}^{2}$ is a factor of N larger than all other terms, so that in the learned system the entries $J_{ij}$ are only an 𝒪(1/N) perturbation away from being i.i.d. Gaussian with variance $g^{2}/N$. Such a small perturbation might seem negligible, but if it has coherent structure, it can produce 𝒪(1) macroscopic effects, pulling outlier eigenvalues out of the random bulk and reshaping activations and dynamics. This is precisely what we observe, suggesting that the learned weight matrix resembles an i.i.d. Gaussian matrix plus a low-rank correction encoding task-relevant structure. In the random-plus-low-rank connectivity literature, this decomposition is specified by construction, either with the low-rank part independent of the random bulk [68, 69] or correlated with it [70, 71]. In our setting, the decomposition is never specified but instead emerges implicitly from end-to-end training; at β→∞, the learned weights can be understood geometrically as uniform samples from the intersection of the high-dimensional spherical shell corresponding to i.i.d. Gaussian weights with a low-dimensional, nonlinearly curved manifold defined by the constraint that the network solves the task. This difference in how the weights are determined leads to a corresponding difference in the DMFT. In random-plus-low-rank models, the order parameters consist of two-time correlation functions tracking bulk-induced fluctuations together with a finite number of scalar overlaps with the low-rank directions [72]. In our theory, the same bulk correlation functions appear, but the role of the low-rank overlaps is replaced by the non-Gaussian tilting of the single-site measure, which implicitly encodes whatever low-rank structure the task demands. Making this correspondence precise is an interesting direction for future work.

During preparation of this manuscript, Bauer et al. [73] released a preprint on feature learning in RNNs, using a mathematical framework largely shared with both our earlier conference presentation [37] and the present work. Despite this common formalism, their work and ours address different questions and operate in different regimes. Their central question is whether and when weight sharing across timesteps in an RNN matters relative to a deep feedforward network with untied weights, a comparison studied primarily through endpoint-supervised tasks in which input enters at the first timestep and a prediction is read out at the last. No such comparison arises in our setting, where the task depends on RNN activity at all time points, as is typical when training RNNs. Their analytical results are restricted to linear activations, and their numerical validation of the nonlinear theory is limited to a regime where learned representations differ only mildly from the reservoir baseline; linear networks cannot exhibit chaos or generate autonomous dynamics. By contrast, we solve the full nonlinear DMFT and demonstrate phenomena inaccessible in the linear or near-reservoir regime, including chaos suppression, the phase transition to low-dimensional dynamics, and temporal generalization. Finally, no analog of our parameter γ appears in their framework; the degree of feature learning is an implicit consequence of task signal strength rather than a continuously controllable quantity, precluding the kind of systematic exploration of the solution space and comparison to neural data that γ enables here. The two works are thus complementary in focus.

Our finding that networks matching neural data require non-Gaussian single-neuron statistics connects to recent work on statistical models of neural populations. Gaussian models of neural activity, or simple nonlinear transformations thereof, have been surprisingly successful in capturing the statistics of systems such as head-direction cells [7] and hippocampal place fields [8]. In the present setting, however, the reservoir regime is Gaussian and fails to match neural data. Recurrent restructuring provides a systematic, theoretically grounded way to go beyond Gaussian models, with γ continuously controlling the degree of non-Gaussianity. Comparing the single-neuron distributions produced by recurrent restructuring with those measured in systems where firing rates or membrane potentials are known to be strongly non-Gaussian [74, 75] could be a productive direction for future work.

Our theory also makes predictions about variability over the timescale of learning. Once the Langevin dynamics reach equilibrium, the weights continue to explore configurations sampled from the Gibbs distribution. Individual weights and single-neuron responses evolve continuously over the learning time s, but population-level quantities such as the correlation function and network outputs remain stable, up to fluctuations that shrink as $1/\sqrt{N}$. For large networks undergoing noisy plasticity, behavior would thus be stable despite continual turnover of single-neuron activity [55, 76]. This is reminiscent of representational drift, the empirical phenomenon in which the response properties of single neurons change continuously over days and weeks while network-level task performance remains stable [77, 78]. Past work has hypothesized that recurrent dynamics play an important role in determining the relative stability of representations of different stimuli [78]. Characterizing the equilibrium and non-equilibrium dynamics over the learning timescale in our framework, as has been done for feedforward networks [79,80], could allow one to isolate the role of recurrent dynamics in a theoretically precise way.

Our framework opens several directions for further investigation. The compositional and modular representations that emerge when RNNs are trained simultaneously on multiple cognitive tasks [21, 22, 81] clearly require recurrent restructuring, and studying how γ controls the emergence of such structure is a natural next step. Applying the theory across a wider range of motor, decision-making, and cognitive tasks would test whether the picture suggested by our motor-cortex results, that circuits are largely random with varying degrees of task-relevant recurrent structure, extends to other systems.

Finally, our results raise the question of why large neural circuits might tend to operate in a regime where recurrent connectivity is only modestly restructured. A possible answer lies in the biological constraints on learning. Training a readout from a fixed representation is straightforward, requiring only a local learning rule that depends on pre- and postsynaptic activity, and corresponds in our framework to learning the readout weights V while leaving recurrent weights J unchanged. Restructuring recurrent connectivity, by contrast, requires propagating error signals backward through time, a computation that is difficult for a biological circuit to implement [82], perhaps even more so than the spatial credit assignment problem faced by feedforward networks. If the recurrent credit assignment problem is indeed hard for biology, then neural circuits may prefer solutions that reshape random connectivity as little as possible while still achieving adequate performance. This tendency would be especially pronounced in large mammalian circuits, where the high-dimensional parameter space and the relatively low-dimensional nature of behavioral tasks create a vast degeneracy of solutions, many of which require only modest recurrent restructuring, and selection pressure need only find one.

## Supplementary Material

Supplement 1

## Footnotes

2 A subset of the authors presented preliminary results at the 2024 Computational and Systems Neuroscience Conference [37], including the general dynamical mean-field formulation and the analytical solution for linear RNNs.
