# Supplementary material for: Structure, disorder, and dynamics in task-trained recurrent neural circuits
Source: bioRxiv. 2026 May 18:2026.03.02.708943. Preprint. [Version 2] doi: 10.64898/2026.03.02.708943 (PMC13228304; doi:10.64898/2026.03.02.708943)
Supplement: Supplement 1 [file NIHPP2026.03.02.708943v2-supplement-1.pdf]

bioRxiv preprint doi: https://doi.org/10.64898/2026.03.02.708943; this version posted May 18, 2026. The copyright holder for this preprint (which was not certified by peer review) is the author/funder, who has granted bioRxiv a license to display the preprint in perpetuity. It is made available under a CC-BY 4.0 International license.

Supplementary Information

SI.1 Core dynamical mean-field theory SI–1
SI.1.1 Which limit do we analyze? . . . SI–1
SI.1.2 Derivation of the mean-field equations . . . SI–2
SI.1.3 Numerical solution of the DMFT equations . . . SI–6

SI.2 Multi-sequence settings and generalization SI–8
SI.2.1 Multi-sequence (batch) case . . . SI–8
SI.2.2 Generalization . . . SI–9

SI.3 Continuous time, time-translation invariance, and linear networks SI–12
SI.3.1 Continuous-time limit . . . SI–12
SI.3.2 Time-translation-invariant solution . . . SI–13
SI.3.3 Linear time-translation-invariant solution . . . SI–15
SI.3.4 Frequency suppression in nonlinear networks . . . SI–19

SI.4 Comparing notions of effective dimensionality of activity SI–21

SI.5 Sine wave (temporal generalization) task SI–23

SI.6 Reaching task and neural data analysis SI–23

SI.7 p-body generalization of the model SI–25

SI.1 Core dynamical mean-field theory

This appendix derives the dynamical mean-field theory (DMFT) for our task-trained RNN. We begin by specifying the order of limits we analyze (Sec. SI.1.1), then carry out the derivation in discrete time for a single input–output sequence (Sec. SI.1.2), and finally describe the numerical methods used to solve the resulting saddle-point equations (Sec. SI.1.3). Subsequent appendices extend the theory to multiple sequences and generalization (Sec. SI.2), and to the continuous-time, time-translation-invariant, and linear regimes (Sec. SI.3).

SI.1.1 Which limit do we analyze?

As mentioned in the main text, we consider Langevin dynamics of the RNN parameters $\boldsymbol{\Theta}$, with learning time s and noise variance $2/\beta$ (Eq. (4)). We are often interested in the behavior of the system for large s

bioRxiv preprint doi: https://doi.org/10.64898/2026.03.02.708943; this version posted May 18, 2026. The copyright holder for this preprint (which was not certified by peer review) is the author/funder, who has granted bioRxiv a license to display the preprint in perpetuity. It is made available under a CC-BY 4.0 International license.

(train for a long time), large β (inject small amounts of noise), and large N (use a large network). However, these three limits generally do not commute [34, 80]. In the present work, we consider the following order of limits.

1. **Large s limit.** We first take $s \to \infty$. In this limit, $\boldsymbol{\Theta}$ is drawn from the Gibbs distribution $\propto \exp(-\beta E(\boldsymbol{\Theta}))$, which depends on β and N.

2. **Large N limit.** We next take $N \to \infty$. This leads to the DMFT derivation of Sec. SI.1.2.

3. **Large β limit.** Finally, we can optionally take the low-temperature limit $\beta \to \infty$, in which the task is fit perfectly.

While this order of limits captures the equilibrium behavior, it does not capture the learning dynamics over s. We leave an analysis of that regime in the $N \to \infty$ limit to future work. It is also distinct from taking the limit $\beta \to \infty$ before the limit $s \to \infty$, which would correspond to networks trained using noiseless gradient flow [67].

SI.1.2 Derivation of the mean-field equations

We derive the DMFT in discrete time, then take the continuous-time limit in Sec. SI.3.1. We discretize time with step Δt, writing $t_n = n\,\Delta t$ for $n = 1, \ldots, n_T$. The network dynamics read

$$x_i(t_1) = x^0, \tag{SI.1}$$

$$x_i(t_n) = (1-\Delta t)\,x_i(t_{n-1}) + \Delta t\left(\frac{g}{\sqrt{N}}\sum_{j=1}^{N} J_{ij}\,\phi\big(x_j(t_{n-1})\big) + \sum_{a=1}^{D_{\text{in}}} U_{ia}\,I_a(t_{n-1})\right), \quad n \geq 2, \tag{SI.2}$$

and the readout is

$$y_a(t_n) = \frac{1}{N\gamma}\sum_{i=1}^{N} V_{ia}\,\phi\big(x_i(t_n)\big). \tag{SI.3}$$

The energy function is

$$E(\boldsymbol{\Theta}) = \frac{N\gamma^2}{2\,n_T D_{\text{out}}}\sum_{n=1}^{n_T}\sum_{a=1}^{D_{\text{out}}}\big(y_a(t_n) - y_a^\star(t_n)\big)^2 + \frac{1}{2\beta}\Big(\|\boldsymbol{J}\|_F^2 + \|\boldsymbol{U}\|_F^2 + \|\boldsymbol{V}\|_F^2\Big), \tag{SI.4}$$

where $\boldsymbol{\Theta} = \{\boldsymbol{J}, \boldsymbol{U}, \boldsymbol{V}\}$. Since we consider the $s \to \infty$ limit of the Langevin dynamics, we wish to evaluate the partition function for the equilibrium measure of $\boldsymbol{\Theta}$,

$$Z = \int d\boldsymbol{\Theta}\,\exp\big(-\beta\,E(\boldsymbol{\Theta})\big). \tag{SI.5}$$

bioRxiv preprint doi: https://doi.org/10.64898/2026.03.02.708943; this version posted May 18, 2026. The copyright holder for this preprint (which was not certified by peer review) is the author/funder, who has granted bioRxiv a license to display the preprint in perpetuity. It is made available under a CC-BY 4.0 International license.

In the energy, the readout $y_a(t_n)$ depends on $\boldsymbol{\Theta}$ in a complicated, highly nonlinear way through the network dynamics. To facilitate integration over $\boldsymbol{\Theta}$, we use the Martin–Siggia–Rose–De Dominicis–Janssen (MSRDJ) path-integral approach [83–85]. We enforce the network dynamics via delta-function constraints, representing each delta function in its Fourier form with conjugate variables $\hat{x}_i(t_n)$ integrated over $(-\infty, \infty)$, and similarly introduce auxiliary output fields $\hat{y}_a(t_n)$ running over $(-i\infty, i\infty)$ for the readout constraint. Averaging over the i.i.d. Gaussian weights $\boldsymbol{\Theta}$ then gives

$$\begin{aligned} Z = \Bigg\langle \int \mathcal{D}\boldsymbol{x} \int \mathcal{D}\hat{\boldsymbol{x}} \int \mathcal{D}\boldsymbol{y} \int \mathcal{D}\hat{\boldsymbol{y}} \, \exp\Bigg\{ & i \sum_{i=1}^{N} \hat{x}_i(t_1)\, x^0 \\ & + i \sum_{i=1}^{N} \sum_{n=2}^{n_T} \hat{x}_i(t_n) \left[x_i(t_n) - (1-\Delta t)\, x_i(t_{n-1}) - \Delta t \left(\frac{g}{\sqrt{N}} \sum_{j=1}^{N} J_{ij} \phi(x_j(t_{n-1})) + \sum_{a=1}^{D_{\text{in}}} U_{ia} I_a(t_{n-1}) \right) \right] \\ & - N \sum_{a=1}^{D_{\text{out}}} \sum_{n=1}^{n_T} \Delta t \, \hat{y}_a(t_n) \left(y_a(t_n) - \frac{1}{N\gamma} \sum_{i=1}^{N} V_{ia} \phi(x_i(t_n)) \right) \\ & - \frac{\beta N \gamma^2}{2\, n_T D_{\text{out}}} \sum_{a=1}^{D_{\text{out}}} \sum_{n=1}^{n_T} (y_a(t_n) - y_a^\star(t_n))^2 \Bigg\} \Bigg\rangle_{\boldsymbol{\Theta}} . \end{aligned} \tag{SI.6}$$

The average over $\boldsymbol{J}$ generates a term quartic in the activations $\phi_i(t_n)$, in which neurons are coupled through the empirical correlation $(1/N) \sum_i \phi_i(t_n)\, \phi_i(t_{n'})$. We decouple this term by promoting the empirical correlation to an order parameter $C(t_n, t_{n'})$, with conjugate field $\hat{C}(t_n, t_{n'})$ enforcing the definition $C(t_n, t_{n'}) = (1/N) \sum_i \phi_i(t_n)\, \phi_i(t_{n'})$ via a Fourier representation of the delta function. We collect these into $n_T \times n_T$ matrices $\boldsymbol{C}$ (with elements integrated along the real axis) and $\hat{\boldsymbol{C}}$ (along the imaginary axis). The partition function then takes the form

$$\begin{aligned} Z = \int \mathcal{D}\boldsymbol{C}\, \mathcal{D}\hat{\boldsymbol{C}}\, \mathcal{D}\boldsymbol{y}\, \mathcal{D}\hat{\boldsymbol{y}} \, \exp\Bigg\{ & -\frac{N}{2} \sum_{n,n'=1}^{n_T} \Delta t^2\, \hat{C}(t_n, t_{n'})\, C(t_n, t_{n'}) - N \sum_{n=1}^{n_T} \Delta t \sum_{a=1}^{D_{\text{out}}} \hat{y}_a(t_n)\, y_a(t_n) \\ & + \frac{N}{2\gamma^2} \sum_{n,n'=1}^{n_T} \Delta t^2 \sum_{a=1}^{D_{\text{out}}} \hat{y}_a(t_n)\, C(t_n, t_{n'})\, \hat{y}_a(t_{n'}) - \frac{N\beta\gamma^2}{2\, n_T D_{\text{out}}} \sum_{a=1}^{D_{\text{out}}} \sum_{n=1}^{n_T} \big(y_a(t_n) - y_a^\star(t_n) \big)^2 \\ & + N \log W(\boldsymbol{C}, \hat{\boldsymbol{C}}) \Bigg\}, \end{aligned} \tag{SI.7}$$

where the single-neuron generating functional $W(\boldsymbol{C}, \hat{\boldsymbol{C}})$ is defined by

$$W(\boldsymbol{C}, \hat{\boldsymbol{C}}) = \left\langle \exp\left(\tfrac{\Delta t^2}{2}\, \boldsymbol{\phi}^\top \hat{\boldsymbol{C}}\, \boldsymbol{\phi} \right) \right\rangle_{\boldsymbol{\eta} \sim \mathcal{N}(\boldsymbol{0},\, g^2 \boldsymbol{C} + \boldsymbol{C}^I)} . \tag{SI.8}$$

bioRxiv preprint doi: https://doi.org/10.64898/2026.03.02.708943; this version posted May 18, 2026. The copyright holder for this preprint (which was not certified by peer review) is the author/funder, who has granted bioRxiv a license to display the preprint in perpetuity. It is made available under a CC-BY 4.0 International license.

Here $\boldsymbol{\phi} = (\phi(t_1), \ldots, \phi(t_{n_T}))$ is the n_T-dimensional vector of activations generated by the single-neuron dynamics

$$x(t_1) = x^0, \tag{SI.9}$$

$$x(t_n) = (1 - \Delta t)\, x(t_{n-1}) + \Delta t\, \eta(t_{n-1}), \quad n \geq 2, \tag{SI.10}$$

with $\phi(t_n) = \phi(x(t_n))$, and $\eta(t_n)$ is a Gaussian process with covariance $\langle \eta(t_n)\, \eta(t_{n'}) \rangle = g^2 C(t_n, t_{n'}) + C^I(t_n, t_{n'})$. The expectation in Eq. (SI.8) is taken over realizations of $\eta(t_n)$.

Reading off the action $\mathcal{S}(\boldsymbol{C}, \hat{\boldsymbol{C}}, \boldsymbol{Y}, \hat{\boldsymbol{Y}})$ such that $Z = \int \mathcal{D}\boldsymbol{C}\, \mathcal{D}\hat{\boldsymbol{C}}\, \mathcal{D}\boldsymbol{Y}\, \mathcal{D}\hat{\boldsymbol{Y}}\, \exp(-N\, \mathcal{S})$, we have

$$\mathcal{S}(\boldsymbol{C}, \hat{\boldsymbol{C}}, \boldsymbol{Y}, \hat{\boldsymbol{Y}}) = \frac{\Delta t^2}{2}\, \mathrm{tr}\, \boldsymbol{C}\hat{\boldsymbol{C}} + \Delta t\, \mathrm{tr}\, \boldsymbol{Y}^\top \hat{\boldsymbol{Y}} - \frac{\Delta t^2}{2\gamma^2}\, \mathrm{tr}\, \hat{\boldsymbol{Y}}^\top \boldsymbol{C} \hat{\boldsymbol{Y}} + \frac{\beta \gamma^2}{2\, n_T D_{\mathrm{out}}} \|\boldsymbol{Y} - \boldsymbol{Y}^\star\|_F^2 - \log W(\boldsymbol{C}, \hat{\boldsymbol{C}}), \tag{SI.11}$$

where $\boldsymbol{Y}$ and $\hat{\boldsymbol{Y}}$ are $n_T \times D_{\mathrm{out}}$ matrices with elements $y_a(t_n)$ and $\hat{y}_a(t_n)$ respectively. The action is quadratic in $\boldsymbol{Y}$ and $\hat{\boldsymbol{Y}}$, so their saddle-point evaluations correspond to exact Gaussian integrations. Evaluating first the saddle point for $\hat{\boldsymbol{Y}}$ gives

$$\mathcal{S}(\boldsymbol{C}, \hat{\boldsymbol{C}}, \boldsymbol{Y}) = \frac{\Delta t^2}{2}\, \mathrm{tr}\, \boldsymbol{C}\hat{\boldsymbol{C}} + \frac{\gamma^2}{2}\, \mathrm{tr}\, \boldsymbol{Y}^\top \boldsymbol{C}^{-1} \boldsymbol{Y} + \frac{\beta \gamma^2}{2\, n_T D_{\mathrm{out}}} \|\boldsymbol{Y} - \boldsymbol{Y}^\star\|_F^2 - \log W(\boldsymbol{C}, \hat{\boldsymbol{C}}). \tag{SI.12}$$

Evaluating next the saddle point for $\boldsymbol{Y}$ yields the output

$$\boldsymbol{Y} = \boldsymbol{C} \left(\boldsymbol{C} + \frac{n_T D_{\mathrm{out}}}{\beta}\, \boldsymbol{I}_{n_T} \right)^{-1} \boldsymbol{Y}^\star, \tag{SI.13}$$

which, in the $\beta \to \infty$ limit, reduces to $\boldsymbol{Y} = \boldsymbol{Y}^\star$, provided that $\boldsymbol{C}$ is invertible.

Substituting Eq. (SI.13) back and simplifying, the action reduces to

$$\mathcal{S}(\boldsymbol{C}, \hat{\boldsymbol{C}}) = \frac{\gamma^2}{2}\, \mathrm{tr} \left(\left(\boldsymbol{C} + \frac{n_T D_{\mathrm{out}}}{\beta}\, \boldsymbol{I}_{n_T} \right)^{-1} \boldsymbol{C}^y \right) + \frac{\Delta t^2}{2}\, \mathrm{tr}\, \boldsymbol{C}\hat{\boldsymbol{C}} - \log W(\boldsymbol{C}, \hat{\boldsymbol{C}}), \tag{SI.14}$$

where $\boldsymbol{C}^y$ is the $n_T \times n_T$ target correlation matrix with elements

$$C^y(t_n, t_{n'}) = \sum_{a=1}^{D_{\mathrm{out}}} y_a^\star(t_n)\, y_a^\star(t_{n'}). \tag{SI.15}$$

Recall that elements of $\hat{\boldsymbol{C}}$ run over $(-i\infty, i\infty)$, and those of $\boldsymbol{C}$ run over $(-\infty, \infty)$. For the saddle-point approximation to be valid, the action must therefore be maximized in $\hat{\boldsymbol{C}}$ and minimized in $\boldsymbol{C}$. The

bioRxiv preprint doi: https://doi.org/10.64898/2026.03.02.708943; this version posted May 18, 2026. The copyright holder for this preprint (which was not certified by peer review) is the author/funder, who has granted bioRxiv a license to display the preprint in perpetuity. It is made available under a CC-BY 4.0 International license.

stationary point of interest is therefore a genuine saddle, with directions of both negative and positive curvature, and solving for this point numerically is nontrivial (Sec. SI.1.3).

We can make the structure of this action more transparent by writing

$$\mathcal{S}(\boldsymbol{C}) = \mathcal{S}_{\mathrm{SCS}}(\boldsymbol{C}) + \frac{\gamma^2}{2}\,\mathrm{tr}\left(\left(\boldsymbol{C} + \frac{n_T D_{\mathrm{out}}}{\beta}\,\boldsymbol{I}_{n_T}\right)^{-1}\boldsymbol{C}^y\right), \tag{SI.16}$$

where

$$\mathcal{S}_{\mathrm{SCS}}(\boldsymbol{C}) = \sup_{\hat{\boldsymbol{C}}}\left\{\tfrac{\Delta t^2}{2}\ \mathrm{tr}\ \boldsymbol{C}\hat{\boldsymbol{C}} - \log W(\boldsymbol{C},\hat{\boldsymbol{C}})\right\}. \tag{SI.17}$$

The first term in $\mathcal{S}(\boldsymbol{C})$, $\mathcal{S}_{\mathrm{SCS}}(\boldsymbol{C})$, encodes the statistics of the random, unstructured network inherited from the SCS theory. The second is the learning term, which penalizes misalignment between the network's temporal correlations and those of the target.

Self-consistency and the tilted measure. The saddle-point condition $\partial\mathcal{S}/\partial\hat{C}(t_n,t_{n'}) = 0$ yields a self-consistency equation that determines $C(t_n,t_{n'})$ in terms of the single-site measure defined by $W(\boldsymbol{C},\hat{\boldsymbol{C}})$. Concretely, $W(\boldsymbol{C},\hat{\boldsymbol{C}})$ defines a tilted probability measure on input currents,

$$P_{\mathrm{tilt}}(\boldsymbol{\eta}) \propto P_0(\boldsymbol{\eta})\ \exp\left(\tfrac{\Delta t^2}{2}\ \boldsymbol{\phi}[\boldsymbol{\eta}]^\top\hat{\boldsymbol{C}}\,\boldsymbol{\phi}[\boldsymbol{\eta}]\right), \tag{SI.18}$$

where $P_0(\boldsymbol{\eta}) = \mathcal{N}(\boldsymbol{\eta};\,\mathbf{0},\,g^2\boldsymbol{C} + \boldsymbol{C}^I)$ is the untilted Gaussian measure and $\boldsymbol{\phi}$ is the activation profile obtained by passing $\boldsymbol{\eta}$ through the single-neuron dynamics (SI.9) and applying the nonlinearity (the induced distribution over activations takes the form $P_{\mathrm{tilt}}(\boldsymbol{\phi}) \propto P_0(\boldsymbol{\phi})\ \exp\left(\frac{\Delta t^2}{2}\ \boldsymbol{\phi}^\top\hat{\boldsymbol{C}}\,\boldsymbol{\phi}\right)$, where $P_0(\boldsymbol{\phi}) = \int d\boldsymbol{\eta} P_0(\boldsymbol{\eta})\delta(\boldsymbol{\phi} - \boldsymbol{\eta})$, noting that the tilting factor is constant where the integrand is nonzero). The self-consistency condition requires that the correlation function computed under the tilted measure reproduces the order parameter,

$$C(t_n,t_{n'}) = \frac{\left\langle \phi(t_n)\,\phi(t_{n'})\ \exp\left(\frac{\Delta t^2}{2}\ \boldsymbol{\phi}^\top\hat{\boldsymbol{C}}\,\boldsymbol{\phi}\right)\right\rangle_{\boldsymbol{\eta}\sim\mathcal{N}(\mathbf{0},\,g^2\boldsymbol{C}+\boldsymbol{C}^I)}}{\left\langle \exp\left(\frac{\Delta t^2}{2}\ \boldsymbol{\phi}^\top\hat{\boldsymbol{C}}\,\boldsymbol{\phi}\right)\right\rangle_{\boldsymbol{\eta}\sim\mathcal{N}(\mathbf{0},\,g^2\boldsymbol{C}+\boldsymbol{C}^I)}}. \tag{SI.19}$$

When $\hat{\boldsymbol{C}} = \mathbf{0}$, as occurs in the reservoir limit $\gamma \to 0^+$, the tilting factor reduces to unity, the input current $\boldsymbol{\eta}$ is simply Gaussian, and Eq. (SI.19) recovers the classical SCS self-consistency equation. When $\hat{\boldsymbol{C}} \neq 0$, the exponential tilting biases the population toward neurons whose input currents produce activations aligned with the structure encoded in $\hat{\boldsymbol{C}}$, driving single-neuron statistics away from Gaussianity in a task-dependent manner. This tilted measure and the associated self-consistency equation will reappear, in progressively generalized forms, in the multi-sequence and generalization settings of Sec. SI.2.

One statistical framework that provides conceptual insight into the adapted single site distribution (and also provides a practical sampling scheme for numerics) is *importance sampling*, where one reinterprets

bioRxiv preprint doi: https://doi.org/10.64898/2026.03.02.708943; this version posted May 18, 2026. The copyright holder for this preprint (which was not certified by peer review) is the author/funder, who has granted bioRxiv a license to display the preprint in perpetuity. It is made available under a CC-BY 4.0 International license.

averages $\langle F(\eta)\rangle_{\eta\sim p(\eta)}$ over a challenging distribution $p(\eta)$ with averages over a simpler distribution $q(\eta)$ as $\left\langle \frac{p(\eta)}{q(\eta)} F(\eta) \right\rangle_{\eta\sim q(\eta)}$ [86]. In our DMFT, we can take $q(\eta)$ to be the Gaussian process, while $p(\eta)$ is the fully non-Gaussian adapted single site distribution. The *importance weight* $\frac{p(\eta)}{q(\eta)}$ is exactly the tilting factor $\exp\left(\frac{1}{2}\int_0^T dt \int_0^T dt' \hat{C}(t,t')\phi(t)\phi(t')\right)$.

SI.1.3 Numerical solution of the DMFT equations

The DMFT equations must be solved numerically for nonlinear $\phi(\cdot)$. We use an alternating iteration procedure to converge to the saddle point $(\boldsymbol{C}, \hat{\boldsymbol{C}})$.

Alternating iteration procedure. We assume access to an automatically differentiable implementation [87] of the action $\mathcal{S}(\boldsymbol{C}, \hat{\boldsymbol{C}})$. We set hyperparameters $(\alpha, \hat{\alpha})$ controlling the update speed for $\boldsymbol{C}$ and $\hat{\boldsymbol{C}}$ respectively, and follow this procedure.

1. Initialize the correlation functions (we use either $\boldsymbol{C} = \boldsymbol{I}$ or $\boldsymbol{C} = \boldsymbol{C}_{\text{SCS}}$) and $\hat{\boldsymbol{C}} = \boldsymbol{0}$.
2. Alternate the following updates until a convergence criterion is met:
   - Iterate $\boldsymbol{C} \leftarrow \boldsymbol{C} - \alpha \frac{\partial \mathcal{S}}{\partial \hat{\boldsymbol{C}}}$ until convergence at fixed $\hat{\boldsymbol{C}}$.
   - Take a single step $\hat{\boldsymbol{C}} \leftarrow \hat{\boldsymbol{C}} - \hat{\alpha} \frac{\partial \mathcal{S}}{\partial \boldsymbol{C}}$.
3. Return the final $(\boldsymbol{C}, \hat{\boldsymbol{C}})$.

We find that this scheme is significantly more stable than an algorithm that attempts to exactly compute the Legendre transform $\sup_{\hat{\boldsymbol{C}}} \mathcal{S}(\boldsymbol{C}, \hat{\boldsymbol{C}})$ before updating $\boldsymbol{C}$. To understand why, we next discuss a general perspective on the saddle-point solver in terms of preconditioned gradient flow.

A preconditioned flow perspective. To interpret and compare iteration schemes, consider a general preconditioned gradient flow on the DMFT action. Let ς represent a flow time and $\boldsymbol{P}$ a real 2×2 preconditioner matrix. The dynamics are

$$\frac{d}{d\varsigma}\begin{pmatrix} \boldsymbol{C} \\ \hat{\boldsymbol{C}} \end{pmatrix} = -\boldsymbol{P}\begin{pmatrix} \frac{\partial \mathcal{S}}{\partial \boldsymbol{C}} \\ \frac{\partial \mathcal{S}}{\partial \hat{\boldsymbol{C}}} \end{pmatrix}. \tag{SI.20}$$

The two natural choices are a diagonal preconditioner $\boldsymbol{P}_{\text{diag}}$, which uses $\partial\mathcal{S}/\partial\boldsymbol{C}$ to update $\boldsymbol{C}$ and $\partial\mathcal{S}/\partial\hat{\boldsymbol{C}}$ to update $\hat{\boldsymbol{C}}$; and an off-diagonal preconditioner $\boldsymbol{P}_{\text{off-diag}}$, which uses $\partial\mathcal{S}/\partial\hat{\boldsymbol{C}}$ to update $\boldsymbol{C}$ and $\partial\mathcal{S}/\partial\boldsymbol{C}$

bioRxiv preprint doi: https://doi.org/10.64898/2026.03.02.708943; this version posted May 18, 2026. The copyright holder for this preprint (which was not certified by peer review) is the author/funder, who has granted bioRxiv a license to display the preprint in perpetuity. It is made available under a CC-BY 4.0 International license.

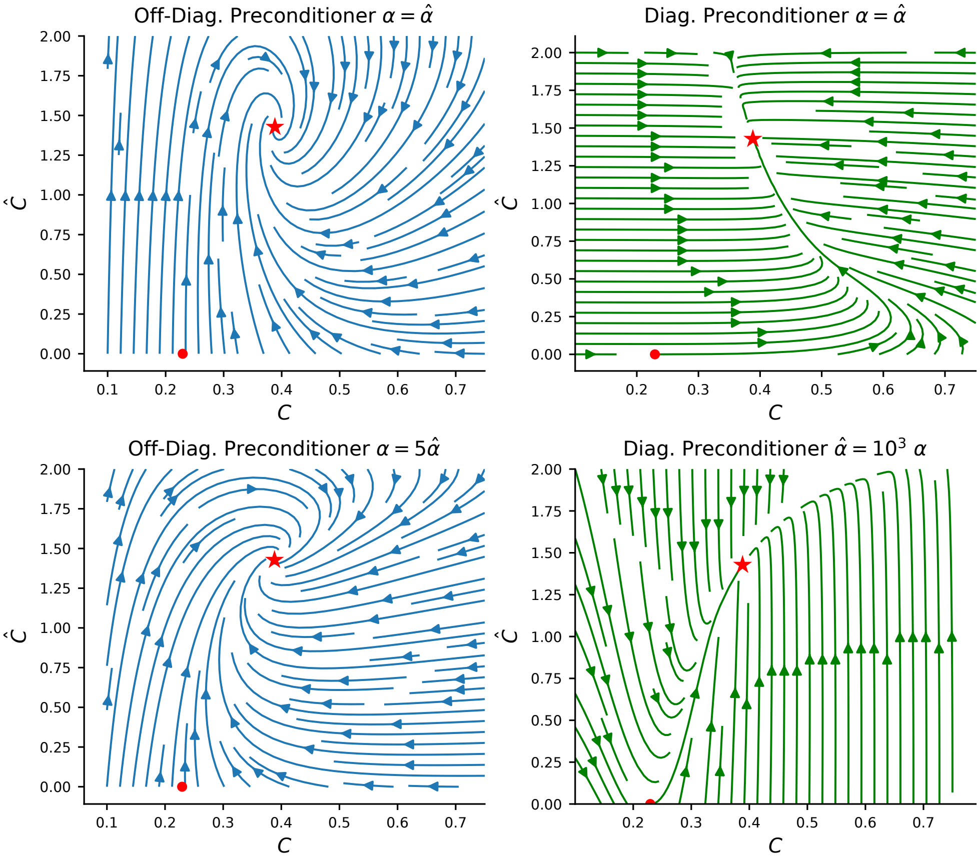

Figure SI.1: Flow field for preconditioned gradient dynamics for a single frequency ω in the time-translation-invariant linear RNN, with $(g, \gamma, \omega) = (0.9, 0.5, 2)$. In all cases, the dynamics flow from the SCS solution (red dot) to the DMFT saddle point (red star). The off-diagonal dynamics are better behaved at comparable step sizes $\alpha \approx \hat{\alpha}$, exhibiting mild spiraling near the saddle point. Monotonic convergence in both C and $\hat{C}$ can be achieved in the off-diagonal scheme with $\alpha/\hat{\alpha} \approx 5$, whereas the diagonal scheme requires $\hat{\alpha}/\alpha \approx 10^3$.

to update $\hat{\boldsymbol{C}}$,

$$\boldsymbol{P}_{\text{diag}} = \begin{pmatrix} \alpha & 0 \\ 0 & -\hat{\alpha} \end{pmatrix}, \qquad \boldsymbol{P}_{\text{off-diag}} = \begin{pmatrix} 0 & \alpha \\ \hat{\alpha} & 0 \end{pmatrix}. \tag{SI.21}$$

Both choices would in principle converge to the correct saddle point. However, the off-diagonal preconditioner (which is essentially our iterative algorithm) is significantly more numerically stable for non-infinitesimal step sizes. Fig. SI.1 illustrates the convergence dynamics for these two preconditioners in the time-translation-invariant linear RNN setting (Sec. SI.3.3).

Computing the differentiable action. We compute $\mathcal{S}(\boldsymbol{C}, \hat{\boldsymbol{C}})$ using an importance-sampling scheme that is compatible with automatic differentiation libraries such as PyTorch [88] and JAX [87]. The procedure is as follows.

bioRxiv preprint doi: https://doi.org/10.64898/2026.03.02.708943; this version posted May 18, 2026. The copyright holder for this preprint (which was not certified by peer review) is the author/funder, who has granted bioRxiv a license to display the preprint in perpetuity. It is made available under a CC-BY 4.0 International license.

1. Perform a Cholesky decomposition of $\boldsymbol{C}$ to obtain a square root $\boldsymbol{C}^{1/2}$.
2. Sample M random Gaussian vectors $\boldsymbol{\eta}_m = \boldsymbol{C}^{1/2}\boldsymbol{z}_m$ where $\boldsymbol{z}_m \sim \mathcal{N}(0, \boldsymbol{I}_{n_T\times n_T})$.
3. Integrate the difference equation (Eq. (SI.9)) to compute $\boldsymbol{x}_m$ from $\boldsymbol{\eta}_m$.
4. Compute the firing-rate vector $\boldsymbol{\phi}_m = \phi(\boldsymbol{x}_m)$.
5. Estimate the single-site partition function $W(\boldsymbol{C}, \hat{\boldsymbol{C}}) = \frac{1}{M}\sum_{m=1}^{M} \exp\left(\frac{\Delta t^2}{2}\, \boldsymbol{\phi}_m^\top \hat{\boldsymbol{C}}\, \boldsymbol{\phi}_m\right)$.
6. Compute the action $\mathcal{S}(\boldsymbol{C}, \hat{\boldsymbol{C}}) = \frac{\gamma^2}{2}\ \mathrm{tr}\ \boldsymbol{C}^y \boldsymbol{C}^{-1} + \frac{\Delta t^2}{2}\ \mathrm{tr}\ \hat{\boldsymbol{C}}\,\boldsymbol{C} - \log W$.

In the time-translation-invariant regime, the Cholesky decomposition can be replaced by Fourier transforms, reducing computational complexity from $\mathcal{O}(n_T^3)$ to $\mathcal{O}(n_T \log n_T)$; the resulting specialized solver for nonlinear networks is described in Sec. SI.3.2.

SI.2 Multi-sequence settings and generalization

The derivation in Sec. SI.1.2 considered a single input–output sequence. We now extend it to a batch of sequences (Sec. SI.2.1) and to generalization on unsupervised sequences or time intervals (Sec. SI.2.2). Throughout, the structure of the action and self-consistency equations carries over with minimal modification; we use parallel notation to make the correspondence explicit.

SI.2.1 Multi-sequence (batch) case

We generalize to a batch of B input–output pairs, consisting of input sequences $I_a^\mu(t_n)$, target outputs $y_a^{\star\mu}(t_n)$, and initial conditions $x^{0\mu}$, where $\mu = 1, \ldots, B$ indexes the sequence.

Energy and order parameters. The MSE term in the energy $E(\boldsymbol{\Theta})$ becomes an average over sequences as well as over time and output channels,

$$\frac{N\gamma^2}{2\, n_T D_{\mathrm{out}} B} \sum_{\mu=1}^{B} \sum_{n=1}^{n_T} \sum_{a=1}^{D_{\mathrm{out}}} \left(y_a^\mu(t_n) - y_a^{\star\mu}(t_n)\right)^2. \tag{SI.22}$$

All dynamical variables acquire a sequence index μ, and the order parameters are promoted to $C^{\mu\nu}(t_n, t_{n'})$ and $\hat{C}^{\mu\nu}(t_n, t_{n'})$, which carry both temporal and batch indices. To handle these enlarged objects compactly, we introduce Fraktur notation (where $\mathfrak{C}$ is the Fraktur version of C, etc.). We write $\mathfrak{C}$ and $\hat{\mathfrak{C}}$ for the full $n_T B \times n_T B$ matrices obtained by treating the pair (μ, t_n) as a single composite index. We similarly write $\mathfrak{Y}$ and $\mathfrak{Y}^\star$ for the output and target matrices, and $\mathfrak{C}^y$ for the target correlation matrix.

bioRxiv preprint doi: https://doi.org/10.64898/2026.03.02.708943; this version posted May 18, 2026. The copyright holder for this preprint (which was not certified by peer review) is the author/funder, who has granted bioRxiv a license to display the preprint in perpetuity. It is made available under a CC-BY 4.0 International license.

Derivation and action. The derivation proceeds exactly as in Sec. SI.1.2, with all traces and inverses now acting on the enlarged $n_T B \times n_T B$ matrices. The learning term $\frac{\gamma^2}{2}\,\mathrm{tr}(\mathfrak{C}^{-1}\mathfrak{C}^y)$ is structurally identical to its single-sequence counterpart, simply promoted to the larger matrices. The only meaningful change appears in the generating functional $W(\mathfrak{C}, \hat{\mathfrak{C}})$, and thus in $\mathcal{S}_{\mathrm{SCS}}(\mathfrak{C})$. Because time does not propagate along the batch dimension, the single-site dynamics consist of B independent trajectories, each evolving from its own initial condition $x^{0\mu}$.

The saddle-point equations for $\mathfrak{Y}$ and $\hat{\mathfrak{Y}}$ are solved as before. The output is

$$\mathfrak{Y} = \mathfrak{C}\left(\mathfrak{C} + \frac{n_T D_{\mathrm{out}} B}{\beta}\,\boldsymbol{I}_{n_T B}\right)^{-1}\mathfrak{Y}^{\star}, \tag{SI.23}$$

which is the direct analog of Eq. (SI.13), with Fraktur matrices replacing their single-sequence counterparts and the regularization factor reflecting the larger number of supervised data points. After substitution, the action is

$$\mathcal{S}(\mathfrak{C}) = \mathcal{S}_{\mathrm{SCS}}(\mathfrak{C}) + \frac{\gamma^2}{2}\,\mathrm{tr}\left(\left(\mathfrak{C} + \frac{n_T D_{\mathrm{out}} B}{\beta}\,\boldsymbol{I}_{n_T B}\right)^{-1}\mathfrak{C}^y\right), \tag{SI.24}$$

$$\mathcal{S}_{\mathrm{SCS}}(\mathfrak{C}) = \sup_{\hat{\mathfrak{C}}}\left\{\tfrac{\Delta t^2}{2}\,\mathrm{tr}\,\mathfrak{C}\,\hat{\mathfrak{C}} - \log W(\mathfrak{C}, \hat{\mathfrak{C}})\right\}, \tag{SI.25}$$

which is the direct analog of Eqs. (SI.16)–(SI.17).

Self-consistency. The self-consistency equation likewise generalizes in the expected way. The untilted Gaussian process is now sampled from the full $n_T B \times n_T B$ covariance $g^2\mathfrak{C} + \mathfrak{C}^I$, the tilting is governed by $\hat{\mathfrak{C}}$, and the self-consistency condition reads

$$C^{\mu\nu}(t_n, t_{n'}) = \frac{\left\langle \phi^{\mu}(t_n)\,\phi^{\nu}(t_{n'})\,\exp\!\left(\frac{\Delta t^2}{2}\,\boldsymbol{\phi}^{\top}\hat{\mathfrak{C}}\,\boldsymbol{\phi}\right)\right\rangle_{\boldsymbol{\eta}\sim\mathcal{N}(\mathbf{0},\,g^2\mathfrak{C}+\mathfrak{C}^I)}}{\left\langle \exp\!\left(\frac{\Delta t^2}{2}\,\boldsymbol{\phi}^{\top}\hat{\mathfrak{C}}\,\boldsymbol{\phi}\right)\right\rangle_{\boldsymbol{\eta}\sim\mathcal{N}(\mathbf{0},\,g^2\mathfrak{C}+\mathfrak{C}^I)}}, \tag{SI.26}$$

where $\boldsymbol{\phi}$ now denotes the concatenation of activations $\boldsymbol{\phi}^{\mu}$ across all B sequences. This is the direct analog of Eq. (SI.19), with the single-sequence measure replaced by the batched one.

SI.2.2 Generalization

The DMFT developed above characterizes the dynamics of the learned network over the sequences at which it was supervised. We now extend the framework to *generalization*, asking what the network dynamics look like on unsupervised (held-out) sequences or time intervals.

bioRxiv preprint doi: https://doi.org/10.64898/2026.03.02.708943; this version posted May 18, 2026. The copyright holder for this preprint (which was not certified by peer review) is the author/funder, who has granted bioRxiv a license to display the preprint in perpetuity. It is made available under a CC-BY 4.0 International license.

Setup. We partition the B sequences into a training set $\mathcal{T} \subset \{1, \ldots, B\}$ and a test set $\mathcal{T}^c = \{1, \ldots, B\} \setminus \mathcal{T}$. The energy $E^{\mathrm{tr}}(\mathbf{\Theta})$ includes contributions only from training sequences, with the MSE in Eq. (SI.22) averaged over $\mu \in \mathcal{T}$ rather than over all B. We write the corresponding partition function as $Z^{\mathrm{tr}} = \int d\mathbf{\Theta}\, \exp(-\beta\, E^{\mathrm{tr}}(\mathbf{\Theta}))$. Evaluating Z^{tr} via the procedure of the preceding sections yields the training DMFT, which determines the train–train block of the order parameters $\mathfrak{C}^{\mathrm{tr}}$ and $\hat{\mathfrak{C}}^{\mathrm{tr}}$.

To compute the dynamical order parameters on the test sequences, we average a separate path integral over the Gibbs measure induced by training,

$$Z^{\mathrm{te}} = \int d\mathbf{\Theta}\, \frac{\exp(-\beta\, E^{\mathrm{tr}}(\mathbf{\Theta}))}{Z^{\mathrm{tr}}}\, Z^{\mathrm{te}}(\mathbf{\Theta}), \tag{SI.27}$$

where $Z^{\mathrm{te}}(\mathbf{\Theta})$ is the path integral that enforces the network dynamics on the test sequences for a given set of weights $\mathbf{\Theta}$, without any squared-error term. Concretely, $Z^{\mathrm{te}}(\mathbf{\Theta})$ takes the form

$$Z^{\mathrm{te}}(\mathbf{\Theta}) = \int \mathcal{D}\boldsymbol{x}^{\mathrm{te}}\, \mathcal{D}\hat{\boldsymbol{x}}^{\mathrm{te}}\, \exp\Bigg\{ i \sum_{\mu \in \mathcal{T}^c} \sum_{i=1}^{N} \hat{x}_i^{\mu}(t_1)\, x^{0\mu} + i \sum_{\mu \in \mathcal{T}^c} \sum_{i=1}^{N} \sum_{n=2}^{n_T} \hat{x}_i^{\mu}(t_n) \Big[x_i^{\mu}(t_n) - (1-\Delta t)\, x_i^{\mu}(t_{n-1}) - \Delta t \Big(\frac{g}{\sqrt{N}} \sum_{j=1}^{N} J_{ij}\, \phi(x_j^{\mu}(t_{n-1})) + \sum_{a=1}^{D_{\mathrm{in}}} U_{ia}\, I_a^{\mu}(t_{n-1}) \Big) \Big] \Bigg\}. \tag{SI.28}$$

When we average $Z^{\mathrm{te}}(\mathbf{\Theta})$ over the training Gibbs measure and introduce order parameters for the combined (train, test) system, the Fraktur matrices $\mathfrak{C}$, $\hat{\mathfrak{C}}$, $\mathfrak{Y}$, and $\hat{\mathfrak{Y}}$ carry indices ranging over all B sequences, both train and test. We proceed with the saddle-point calculation as before. The key simplification comes from the structure of the conjugate variables at the saddle point.

Vanishing of test conjugate variables. At the saddle point, all components of $\hat{\mathfrak{C}}$ and $\hat{\mathfrak{Y}}$ in which at least one index belongs to the test set vanish,

$$\hat{C}^{\mu\nu}(t_n, t_{n'}) = 0 \quad \text{if } \mu \in \mathcal{T}^c \text{ or } \nu \in \mathcal{T}^c, \tag{SI.29}$$

$$\hat{y}_a^{\mu}(t_n) = 0 \quad \text{if } \mu \in \mathcal{T}^c. \tag{SI.30}$$

Only the train–train block $\hat{C}^{\mu\nu}(t_n, t_{n'})$ with $\mu, \nu \in \mathcal{T}$ is nonzero.

This result has a clear physical interpretation. The conjugate variable $\hat{\mathfrak{C}}$ governs the non-Gaussian tilting of the distribution over input currents (Eq. (SI.18)). Test sequences share no error signal, so they cannot tilt one another; the test–test block of $\hat{\mathfrak{C}}$ must therefore vanish. Test sequences also play no role in the training objective, so they cannot influence the tilting of training sequences; the train–test and test–train blocks must vanish as well. The only sequences that interact through the tilting mechanism are

bioRxiv preprint doi: https://doi.org/10.64898/2026.03.02.708943; this version posted May 18, 2026. The copyright holder for this preprint (which was not certified by peer review) is the author/funder, who has granted bioRxiv a license to display the preprint in perpetuity. It is made available under a CC-BY 4.0 International license.

training sequences, which are coupled by virtue of being used to train the same set of weights $\boldsymbol{\Theta}$.

Saddle-point solution. As a consequence of Eq. (SI.29), the training order parameters decouple from the test sequences entirely. The train–train block $\mathfrak{C}^{\mathrm{tr}}$ and its conjugate $\hat{\mathfrak{C}}^{\mathrm{tr}}$ are determined by the same DMFT equations derived in the preceding sections, with no dependence on the test data.

The test-dependent components of $\mathfrak{C}$ are determined by the same tilted single-site measure, but with the tilting restricted to the train–train block. In the single-site problem, the full (train, test) Gaussian process $\boldsymbol{\eta} = (\boldsymbol{\eta}^{\mathrm{tr}}, \boldsymbol{\eta}^{\mathrm{te}})$ is sampled from the $n_T B \times n_T B$ covariance $g^2\mathfrak{C} + \mathfrak{C}^I$, which couples train and test sequences through their shared dependence on the same weights. Each sequence's activations $\boldsymbol{\phi}^\mu$ are obtained by passing the corresponding $\boldsymbol{\eta}^\mu$ through the single-neuron dynamics (SI.9). However, because only the train–train block of $\hat{\mathfrak{C}}$ is nonzero, the tilting involves exclusively the training components of $\boldsymbol{\phi}$. The self-consistency equation for all components of $\mathfrak{C}$ takes the unified form

$$C^{\mu\nu}(t_n, t_{n'}) = \frac{\left\langle \phi^\mu(t_n)\, \phi^\nu(t_{n'})\, \exp\!\left(\frac{\Delta t^2}{2}\, (\boldsymbol{\phi}^{\mathrm{tr}})^\top \hat{\mathfrak{C}}^{\mathrm{tr}}\, \boldsymbol{\phi}^{\mathrm{tr}}\right)\right\rangle_{\boldsymbol{\eta}\sim\mathcal{N}(\mathbf{0},\, g^2\mathfrak{C}+\mathfrak{C}^I)}}{\left\langle \exp\!\left(\frac{\Delta t^2}{2}\, (\boldsymbol{\phi}^{\mathrm{tr}})^\top \hat{\mathfrak{C}}^{\mathrm{tr}}\, \boldsymbol{\phi}^{\mathrm{tr}}\right)\right\rangle_{\boldsymbol{\eta}\sim\mathcal{N}(\mathbf{0},\, g^2\mathfrak{C}+\mathfrak{C}^I)}}, \tag{SI.31}$$

where $\boldsymbol{\phi}^{\mathrm{tr}}$ denotes the concatenation of activations across training sequences and times. Eq. (SI.31) is the direct analog of the batch self-consistency equation (Eq. (SI.26)). The key difference is that the tilting acts only on training activations $\boldsymbol{\phi}^{\mathrm{tr}}$, while the observable $\phi^\mu(t_n)\,\phi^\nu(t_{n'})$ in the numerator may involve test activations. The equation holds for all pairs (μ, ν), whether both are in $\mathcal{T}$, both in $\mathcal{T}^c$, or one in each.

Output. The output formulas likewise parallel those of the preceding sections, with one new element. The train output is given by

$$\mathfrak{Y}^{\mathrm{tr}} = \mathfrak{C}^{\mathrm{tr}} \left(\mathfrak{C}^{\mathrm{tr}} + \frac{n_T D_{\mathrm{out}} |\mathcal{T}|}{\beta}\, \boldsymbol{I}_{n_T|\mathcal{T}|} \right)^{-1} \mathfrak{Y}^{\star\mathrm{tr}}, \tag{SI.32}$$

which is the direct analog of Eqs. (SI.13) and (SI.23), restricted to training sequences. The test output takes the form of a kernel regression, with the cross-covariance between test and train sequences mediating the prediction,

$$\mathfrak{Y}^{\mathrm{te}} = \mathfrak{C}^{\mathrm{te,tr}} \left(\mathfrak{C}^{\mathrm{tr}} + \frac{n_T D_{\mathrm{out}} |\mathcal{T}|}{\beta}\, \boldsymbol{I}_{n_T|\mathcal{T}|} \right)^{-1} \mathfrak{Y}^{\star\mathrm{tr}}, \tag{SI.33}$$

where $\mathfrak{C}^{\mathrm{te,tr}}$ is the $n_T|\mathcal{T}^c| \times n_T|\mathcal{T}|$ cross-covariance block. The only difference from Eq. (SI.32) is that $\mathfrak{C}^{\mathrm{tr}}$ in front of the regularized inverse is replaced by $\mathfrak{C}^{\mathrm{te,tr}}$. The test output is thus determined entirely by the correlation between test and train dynamics and the training targets, with no direct dependence on test targets (which are, by assumption, unavailable).

bioRxiv preprint doi: https://doi.org/10.64898/2026.03.02.708943; this version posted May 18, 2026. The copyright holder for this preprint (which was not certified by peer review) is the author/funder, who has granted bioRxiv a license to display the preprint in perpetuity. It is made available under a CC-BY 4.0 International license.

Application to temporal generalization within a single sequence. The same formalism applies not only to held-out sequences but also to held-out time intervals within a single sequence. In the sine wave generation task of Sec. 2.5, for example, the network is supervised over $[0, T]$ and we ask what it produces for $t > T$. To handle this, we partition the time axis into a supervised block ($\mu = 1$, covering $[0, T]$) and an unsupervised block ($\mu = 2$, covering $[T, T_{\text{tot}}]$), and assign the first to $\mathcal{T}$ and the second to $\mathcal{T}^c$. The formalism then proceeds exactly as above, with one important difference. Unlike the true batch setting, where each sequence starts from an independent initial condition and time does not propagate across sequences, here time *does* propagate across blocks. The state at the end of the supervised window serves as the initial condition for the unsupervised window, coupling the two blocks in the single-site dynamics. This coupling is what allows the framework to predict whether learned temporal structure (such as oscillations) persists beyond the training interval.

The numerical solver consists simply of the $\mathfrak{C}$ iterations with the tilting matrix fixed. That is, $\mathfrak{C}^{\text{tr}}$ is held at its training saddle-point value while the test-dependent components of $\mathfrak{C}$ are iterated.

SI.3 Continuous time, time-translation invariance, and linear networks

In this appendix we specialize the DMFT to settings that admit simplification and, in some cases, closed-form solutions. We first take the continuous-time limit (Sec. SI.3.1), then consider time-translation-invariant (TTI) solutions that decouple the DMFT across Fourier modes (Sec. SI.3.2), and finally solve the TTI equations analytically for linear networks (Sec. SI.3.3). The appendix concludes with a comparison between linear and nonlinear networks (Sec. SI.3.4), highlighting a qualitatively new phenomenon enabled by the nonlinearity, namely the suppression of unused frequencies.

SI.3.1 Continuous-time limit

The continuous-time limit is obtained by taking $\Delta t \to 0$ with $T = n_T \Delta t$ held fixed. Discrete sums $\sum_n \Delta t\,(\cdots)$ become integrals $\int_0^T dt\,(\cdots)$. The matrices $\boldsymbol{C}$, $\hat{\boldsymbol{C}}$, and $\boldsymbol{C}^y$ become continuous-time kernels $C(t,t')$, $\hat{C}(t,t')$, and $C^y(t,t')$. The matrix trace $\frac{\Delta t^2}{2}\ \mathrm{tr}\ \boldsymbol{C}\hat{\boldsymbol{C}}$ becomes $\mathrm{tr}(C\,\hat{C}) = \int_0^T dt \int_0^T dt'\, C(t,t')\,\hat{C}(t',t)$, and the learning term $\frac{\gamma^2}{2}\,\mathrm{tr}((\boldsymbol{C}+\frac{n_T D_{\text{out}}}{\beta}\,\boldsymbol{I}_{n_T})^{-1}\,\boldsymbol{C}^y)$ becomes $\frac{\gamma^2}{2}\int_0^T dt \int_0^T dt'\,(C+\frac{T D_{\text{out}}}{\beta}\,\delta)^{-1}(t,t')\,C^y(t',t)$, where $\delta(t-t')$ is a Dirac delta. The discrete single-neuron dynamics (SI.9) become $(1+\partial_t)\,x(t) = \eta(t)$.

In this limit, the action (SI.16) becomes

$$\mathcal{S}(C) = \mathcal{S}_{\text{SCS}}(C) + \frac{\gamma^2}{2}\,\mathrm{tr}\left(\left(C + \tfrac{T D_{\text{out}}}{\beta}\,\delta\right)^{-1} C^y\right), \tag{SI.34}$$

bioRxiv preprint doi: https://doi.org/10.64898/2026.03.02.708943; this version posted May 18, 2026. The copyright holder for this preprint (which was not certified by peer review) is the author/funder, who has granted bioRxiv a license to display the preprint in perpetuity. It is made available under a CC-BY 4.0 International license.

where $C^y(t,t') = \sum_{a=1}^{D_{\text{out}}} y_a^\star(t)\, y_a^\star(t')$ and

$$\mathcal{S}_{\text{SCS}}(C) = \sup_{\hat{C}} \left\{ \text{tr}(C\,\hat{C}) - \log W(C,\hat{C}) \right\}, \tag{SI.35}$$

with

$$W(C,\hat{C}) = \left\langle \exp\left(\tfrac{1}{2} \int_0^T dt \int_0^T dt'\, \hat{C}(t,t')\, \phi(t)\, \phi(t') \right) \right\rangle_\eta, \tag{SI.36}$$

where $\phi(t) = \phi(x(t))$, $x(t)$ satisfies $(1+\partial_t)\, x(t) = \eta(t)$, and $\eta(t)$ is a Gaussian process with covariance $g^2 C(t,t') + C^I(t,t')$. In the $\beta \to \infty$ limit, the regularized inverse reduces to C^{-1}, the output matches the target exactly (provided C is invertible), and the action simplifies to $\mathcal{S}(C) = \mathcal{S}_{\text{SCS}}(C) + \frac{\gamma^2}{2}\, \text{tr}(C^{-1} C^y)$. This is the action quoted in Eq. (6) of the main text. The self-consistency equation (Eq. (SI.19)) and the generalizations to the batch (Sec. SI.2.1) and held-out (Sec. SI.2.2) settings carry over with the same substitutions.

SI.3.2 Time-translation-invariant solution

A further simplification arises when the system admits a time-translation-invariant (TTI) solution, in which all two-time correlation functions depend only on time differences. Concretely, we assume that the input and target correlations satisfy $C^I(t,t') = C^I(t-t')$ and $C^y(t,t') = C^y(t-t')$, and we seek solutions in which $C(t,t') = C(t-t')$ and $\hat{C}(t,t') = \hat{C}(t-t')$ likewise. We denote $\tau = t - t'$. (Note that time-translation invariance is a property of the correlation functions, not of the activity $x(t)$ itself, which will generally fluctuate over time.) This regime is unable to describe inherently nonstationary scenarios such as temporal generalization beyond a finite time window (Sec. 2.5) or a finite-duration motor or cognitive task (Sec. 2.6). The key benefit of this regime is that it enables analysis in the Fourier domain [18], providing both dramatic reductions in computational complexity and a simple spectral interpretation of the learned representations. Thus, this TTI regime is extremely common in DMFT studies [18, 89, 90].

We adopt the Fourier transform convention for Fourier pair $f(t) \leftrightarrow f(\omega)$ as

$$f(\omega) = \int_{-\infty}^{\infty} dt\, e^{-i\omega t} f(t), \qquad f(t) = \int_{-\infty}^{\infty} \frac{d\omega}{2\pi}\, e^{i\omega t} f(\omega). \tag{SI.37}$$

Under TTI structure, the action decomposes in Fourier space as

$$\begin{aligned} \mathcal{S}(C,\hat{C}) &= \frac{\gamma^2}{4\pi} \int_{-\infty}^{\infty} d\omega\, \frac{C^y(\omega)}{C(\omega)} + \frac{1}{4\pi} \int_{-\infty}^{\infty} d\omega\, C(\omega)\, \hat{C}(\omega) - \ln W(C,\hat{C}), \\ W(C,\hat{C}) &= \left\langle \exp\left(\frac{1}{4\pi} \int_{-\infty}^{\infty} d\omega\, \hat{C}(\omega)\, |\phi(\omega)|^2 \right) \right\rangle_{\boldsymbol{\eta} \sim \mathcal{N}(\mathbf{0},\, \mathbf{diag}(g^2 C + C^I))}, \end{aligned} \tag{SI.38}$$

bioRxiv preprint doi: https://doi.org/10.64898/2026.03.02.708943; this version posted May 18, 2026. The copyright holder for this preprint (which was not certified by peer review) is the author/funder, who has granted bioRxiv a license to display the preprint in perpetuity. It is made available under a CC-BY 4.0 International license.

where $\phi(\omega)$ is the Fourier transform of $\phi(x(t))$ with $x(t)$ generated by the single-neuron dynamics $(1+\partial_t)\,x(t) = \eta(t)$. Since $\eta(t)$ is a stationary Gaussian process, its Fourier components at distinct frequencies are independent, with $\eta(\omega)$ having variance $g^2C(\omega) + C^I(\omega)$.

The saddle-point equations $\partial\mathcal{S}/\partial\hat{C}(\omega) = 0$ and $\partial\mathcal{S}/\partial C(\omega) = 0$ determine $C(\omega)$ and $\hat{C}(\omega)$ self-consistently. The first gives a self-consistency equation for $C(\omega)$ as an expectation under the tilted measure, paralleling Eq. (SI.19),

$$C(\omega) = \frac{\left\langle |\phi(\omega)|^2 \,\exp\left(\frac{1}{4\pi}\int d\omega'\,\hat{C}(\omega')\,|\phi(\omega')|^2\right)\right\rangle_{\boldsymbol{\eta}\sim\mathcal{N}(\mathbf{0},\,\mathbf{diag}(g^2C+C^I))}}{\left\langle \exp\left(\frac{1}{4\pi}\int d\omega'\,\hat{C}(\omega')\,|\phi(\omega')|^2\right)\right\rangle_{\boldsymbol{\eta}\sim\mathcal{N}(\mathbf{0},\,\mathbf{diag}(g^2C+C^I))}}. \tag{SI.39}$$

The second gives $\hat{C}(\omega)$ in terms of $C(\omega)$ and a functional derivative of $\log W(C,\hat{C})$,

$$\hat{C}(\omega) = \gamma^2\,\frac{C^y(\omega)}{C(\omega)^2} + \frac{\partial \log W(C,\hat{C})}{\partial C(\omega)}. \tag{SI.40}$$

Whether these equations couple different frequencies or decouple into independent problems at each ω depends on whether the generating functional $W(C,\hat{C})$ factorizes across frequencies. The tilting factor in $W(C,\hat{C})$ is $\exp(\frac{1}{4\pi}\int d\omega\,\hat{C}(\omega)\,|\phi(\omega)|^2)$, and the expectation is over a Gaussian process with Fourier components $\eta(\omega)$, which are independent across frequencies. If $\phi(\omega)$ were a function only of $\eta(\omega)$ at the same frequency, then $|\phi(\omega)|^2$ would be independent across frequencies under the η-distribution, the exponent would be a sum of independent terms, and $W(C,\hat{C})$ would factorize into a product of single-frequency contributions. Both saddle-point equations would then decouple across ω.

This is exactly what happens in a linear network, where $\phi(\omega) = x(\omega) = \eta(\omega)/(1+i\omega)$, and we exploit it to obtain closed-form solutions that decouple over frequencies ω in Sec. SI.3.3. When $\phi(\cdot)$ is nonlinear, however, $\phi(t) = \phi(x(t))$ is computed pointwise in the time domain, and Fourier-transforming back gives

$$\phi(\omega) = \int dt\, e^{-i\omega t}\,\phi\left(\int_{-\infty}^{\infty}\frac{d\omega'}{2\pi}e^{i\omega' t}x(\omega')\right), \tag{SI.41}$$

which is a complicated nonlinear mixture of $x(\omega)$ at different frequencies. Thus, $W(C,\hat{C})$ does not factorize, and both saddle-point equations couple all frequencies together. This inter-frequency coupling is what enables the suppression of task-irrelevant frequencies discussed in Sec. SI.3.4.

Spectral bias of the learning term. The structure of Eq. (SI.40) is the frequency-domain counterpart of the general (t,t') principle discussed in Sec. 2.2 of the main text, where the learning term $\frac{\gamma^2}{2}\mathrm{tr}(C^{-1}C^y)$ penalizes misalignment between the network's temporal correlations and those of the target. In the TTI setting, this principle becomes particularly transparent. The ratio $C^y(\omega)/C(\omega)^2$ measures how

bioRxiv preprint doi: https://doi.org/10.64898/2026.03.02.708943; this version posted May 18, 2026. The copyright holder for this preprint (which was not certified by peer review) is the author/funder, who has granted bioRxiv a license to display the preprint in perpetuity. It is made available under a CC-BY 4.0 International license.

much each frequency is under-represented in the network activity relative to the target, and appears in the equation for $\hat{C}(\omega)$ with a prefactor γ^2. When $C(\omega)$ already has substantial power at frequency ω, the contribution to $\hat{C}(\omega)$ is small, and the tilting of the single-neuron distribution at that frequency is correspondingly weak. When $C(\omega)$ is small relative to $C^y(\omega)$, the contribution to $\hat{C}(\omega)$ is large, driving substantial restructuring.

This has two immediate consequences. First, networks trained on higher-frequency targets will generally exhibit larger departures from the random-network correlation structure, because the power spectrum of a random network decays at high frequencies, making the mismatch $C^y(\omega)/C(\omega)^2$ large there. Second, networks with larger gain g will (at fixed γ) show smaller departures, because a larger g slows the spectral decay of $C(\omega)$ and reduces the mismatch. Both consequences apply regardless of whether the nonlinearity $\phi(\cdot)$ is the identity (as in the linear theory below) or a saturating function such as $\tanh$. The linear and nonlinear cases differ, however, in whether the saddle-point equations decouple across frequencies; we return to this distinction in Sec. SI.3.4.

TTI numerical solver. To solve the TTI saddle-point equations numerically, we use the alternating iteration procedure described in Sec. SI.1.3, maintaining a one-dimensional grid of values for $C(\omega)$ and $\hat{C}(\omega)$. The action (SI.38) and its gradients are estimated via importance sampling. Rather than storing and manipulating the full $n_T \times n_T$ matrices $\boldsymbol{C}$ and $\hat{\boldsymbol{C}}$, we sample complex Gaussians $\eta(\omega)$ with variance $g^2 C(\omega) + C^I(\omega)$ at each frequency, inverse-Fourier-transform to obtain $\eta(t)$, integrate the single-neuron dynamics to produce $x(t)$, apply the nonlinearity to get $\phi(t)$, and Fourier-transform back to obtain $\phi(\omega)$. The tilting factor $\frac{1}{4\pi}\int d\omega\, \hat{C}(\omega)\, |\phi(\omega)|^2$ is then computed directly in the frequency domain. This reduces computational complexity from $\mathcal{O}(n_T^3)$ (for a Cholesky decomposition) to $\mathcal{O}(n_T \log n_T)$ (for Fourier transforms).

SI.3.3 Linear time-translation-invariant solution

For a linear network with $\phi(x) = x$, the TTI action simplifies considerably. The single-neuron dynamics $(1 + \partial_t)\, x(t) = \eta(t)$ are a linear filter with transfer function $R(\omega) = 1/(1 + i\omega)$, so $x(\omega) = R(\omega)\, \eta(\omega)$ and $\phi(\omega) = x(\omega)$. The generating functional $W(C, \hat{C})$ can then be computed in closed form, reducing the action to an integral over frequencies of a primitive action at each ω,

$$\mathcal{S}(C, \hat{C}) = \frac{1}{4\pi}\int_{-\infty}^{\infty} d\omega \left[\frac{\gamma^2 C^y(\omega)}{C(\omega) + \beta^{-1}} + C(\omega)\hat{C}(\omega) + \log\left(1 - \hat{C}(\omega)|R(\omega)|^2 (g^2 C(\omega) + C^I(\omega))\right)\right]. \tag{SI.42}$$

A crucial simplification relative to the nonlinear case is that this action decouples across frequencies, with the saddle-point equations at each ω forming a closed system independent of other frequencies. This is because the linear activation $\phi(x) = x$ does not mix Fourier modes.

bioRxiv preprint doi: https://doi.org/10.64898/2026.03.02.708943; this version posted May 18, 2026. The copyright holder for this preprint (which was not certified by peer review) is the author/funder, who has granted bioRxiv a license to display the preprint in perpetuity. It is made available under a CC-BY 4.0 International license.

Varying with respect to $C(\omega)$ and $\hat{C}(\omega)$, we obtain

$$\begin{aligned} 1 &= \frac{C(\omega)}{|R(\omega)|^2[g^2C(\omega) + C^I(\omega)]} - C(\omega)\hat{C}(\omega), \\ \hat{C}(\omega) &= \frac{\gamma^2 C^y(\omega)}{[\beta^{-1} + C(\omega)]^2} + g^2|R(\omega)|^2\hat{C}(\omega)[1 + C(\omega)\hat{C}(\omega)]. \end{aligned} \tag{SI.43}$$

In the reservoir limit $\gamma \to 0^+$, we have $\hat{C}(\omega) = 0$, and $C(\omega)$ reduces to

$$C(\omega) = \frac{C^I(\omega)}{1 + \omega^2 - g^2}, \tag{SI.44}$$

using $|R(\omega)|^2 = 1/(1+\omega^2)$. This is the expected result for a random linear reservoir [18, 67].

Analytical solution at $\gamma > 0$. We now solve the coupled equations for $\gamma > 0$ in the $\beta \to \infty$ limit. Defining

$$Z(\omega) = C(\omega)\hat{C}(\omega), \quad Q(\omega) = \frac{C^y(\omega)}{C^I(\omega)}, \quad \text{and} \quad G(\omega) = \frac{C(\omega)}{C^I(\omega)}, \tag{SI.45}$$

we can rewrite the equations as

$$\begin{aligned} Z(\omega) &= \frac{G(\omega)}{|R(\omega)|^2[1 + g^2 G(\omega)]} - 1, \\ Z(\omega) &= \frac{\gamma^2 Q(\omega)}{G(\omega)} + g^2|R(\omega)|^2 Z(\omega)[1 + Z(\omega)]. \end{aligned} \tag{SI.46}$$

Substituting the first equation into the second and simplifying, we obtain the quadratic equation

$$[1 + \omega^2 - g^2 - \gamma^2 g^4 Q(\omega)]\, G(\omega)^2 - [1 + 2\gamma^2 g^2 Q(\omega)]\, G(\omega) - \gamma^2 Q(\omega) = 0. \tag{SI.47}$$

This leads to

$$G(\omega) = \frac{1 + 2\gamma^2 g^2 Q(\omega) + \sqrt{1 + 4\gamma^2(1+\omega^2)Q(\omega)}}{2[1 + \omega^2 - g^2 - \gamma^2 g^4 Q(\omega)]}, \tag{SI.48}$$

which, upon substituting the expressions for $G(\omega)$ and $Q(\omega)$, gives the closed-form solution quoted in the main text (Eq. (7)). This solution is the physical one because it satisfies $\lim_{\gamma\to 0^+} G(\omega) = 1/(1+\omega^2 - g^2)$, matching the random-network limit; the solution with the negated square root yields $\lim_{\gamma\to 0^+} G(\omega) = 0$ and is unphysical.

bioRxiv preprint doi: https://doi.org/10.64898/2026.03.02.708943; this version posted May 18, 2026. The copyright holder for this preprint (which was not certified by peer review) is the author/funder, who has granted bioRxiv a license to display the preprint in perpetuity. It is made available under a CC-BY 4.0 International license.

Finally, we can solve for

$$\hat{C}(\omega) = \frac{\gamma^2 C^y(\omega)\left(C^I(\omega) + g^2 C(\omega)\right)}{C(\omega)^2 C^I(\omega)} \tag{SI.49}$$

which is manifestly non-negative for any γ.

At a conceptual level, this square-root solution closely resembles the solution for the hidden-layer feature kernel of a single-hidden-layer linear Bayesian neural network [44]. In this dynamical setting, however, the assumption of statistical stationarity means that the input and target autocorrelation functions are co-diagonalizable (by the Fourier transform), which is not guaranteed in the feedforward setting, where one must solve a matrix quadratic equation.

Stability condition. The solution (SI.48) is free of poles only if

$$1 + \omega^2 - g^2 - \gamma^2 g^4 Q(\omega) > 0 \tag{SI.50}$$

for all ω, which requires

$$\gamma^2 < \frac{1 + \omega^2 - g^2}{g^4\, Q(\omega)} \tag{SI.51}$$

for all ω.

Interpretation. The ratio $Q(\omega) = C^y(\omega)/C^I(\omega)$ measures the over-representation of a given frequency in the target signal relative to the input. With nonzero γ, the transfer function is reshaped to amplify precisely those frequencies. This is made manifest by the small-γ expansion,

$$G(\omega) = \frac{1}{1+\omega^2 - g^2} + \gamma^2 \left(1 + \frac{g^2}{1+\omega^2-g^2}\right)^2 Q(\omega) + \mathcal{O}(\gamma^4), \tag{SI.52}$$

which shows that to leading order in γ, each frequency is amplified in proportion to $Q(\omega)$.

As a further illustration, consider white-noise input ($C^I(\omega) = 1$) and an Ornstein–Uhlenbeck target ($C^y(\omega) = 1/(1+\omega^2)$). Then the solution simplifies to

$$C(\omega) = \frac{1}{1+\omega^2 - g^2 u}, \qquad u = \frac{1+\sqrt{1+4\gamma^2}}{2}, \tag{SI.53}$$

so that feature learning has the effect of increasing the effective gain of the linear reservoir from g^2 to $g^2 u$. Stability requires $g^2 u < 1$, or equivalently $\gamma^2 < (1-g^2)/g^4$.

bioRxiv preprint doi: https://doi.org/10.64898/2026.03.02.708943; this version posted May 18, 2026. The copyright holder for this preprint (which was not certified by peer review) is the author/funder, who has granted bioRxiv a license to display the preprint in perpetuity. It is made available under a CC-BY 4.0 International license.

Participation ratio. One useful summary statistic that can be computed from the power spectrum is the effective single-neuron inverse timescale, defined in the stationary setting by (see also Sec. SI.4)

$$\mathrm{PR} = \frac{C(0)^2}{\int_{-\infty}^{\infty} d\tau\, C(\tau)^2} = \frac{1}{2\pi} \frac{\left(\int_{-\infty}^{\infty} d\omega\, C(\omega)\right)^2}{\int_{-\infty}^{\infty} d\omega\, C(\omega)^2}. \tag{SI.54}$$

For an Ornstein–Uhlenbeck process with correlation time τ,

$$C^I(\omega) = \frac{1}{1+\tau^2\omega^2}, \tag{SI.55}$$

which has participation ratio $1/\tau$. This is the basic reason why we refer to this measure as an inverse timescale, or rate.

If we drive a linear reservoir network with white noise, the single neurons obey Ornstein–Uhlenbeck statistics, and we have

$$\mathrm{PR} = \sqrt{1-g^2}; \tag{SI.56}$$

the effective timescale thus diverges as $g \to 1^-$. This is consistent with the result that the participation ratio of activity in linear RNNs should vanish as $g \to 1^-$ in Hu and Sompolinsky [46], and also with the results of Bordelon et al. [67].

Closed-form evaluation of the integrals defining the effective rate for a rich network is not so simple. For the sake of tractability, we first consider a case in which the input process is white noise ($C^I(\omega) = 1$), such that the two integrals of interest are

$$2\int_0^{\infty} d\omega \left[\frac{1+2\gamma^2 g^2\, C^y(\omega) + \sqrt{1+4\gamma^2(1+\omega^2)\, C^y(\omega)}}{2[1+\omega^2-g^2-\gamma^2 g^4\, C^y(\omega)]}\right]^k \tag{SI.57}$$

where $k \in \{1,2\}$. What remains is to make an appropriate choice of $C^y(\omega)$ so that the result is interesting while the computation remains tractable. An interesting test case is the Cauchy (or Lorentzian) spectrum

$$C^y(\omega) = \frac{1}{1+10^2(|\omega|-\omega_0)^2}, \tag{SI.58}$$

because one can distinguish the peak at ω_0 from the flat background spectrum of $C^I(\omega) = 1$. However, the resulting integral in general has the form of an elliptic integral.

We therefore resort to numerical evaluation. To do so stably, we make use of the fact that the integrand

bioRxiv preprint doi: https://doi.org/10.64898/2026.03.02.708943; this version posted May 18, 2026. The copyright holder for this preprint (which was not certified by peer review) is the author/funder, who has granted bioRxiv a license to display the preprint in perpetuity. It is made available under a CC-BY 4.0 International license.

can be simplified algebraically to

$$\frac{1 + 2\gamma^2 g^2\, C^y(\omega) + \sqrt{1 + 4\gamma^2 (1+\omega^2)\, C^y(\omega)}}{2[1 + \omega^2 - g^2 - \gamma^2 g^4\, C^y(\omega)]} = \frac{1}{\chi - g^2} \tag{SI.59}$$

where

$$\chi = \frac{2(1+\omega^2)}{1 + \sqrt{1 + 4\gamma^2(1+\omega^2)\, C^y(\omega)}}. \tag{SI.60}$$

This form is stable numerically even when $C^y(\omega)$ is small. The stability condition is that

$$g^4\gamma^2 < (1 + \omega^2 - g^2)[1 + 10^2(\omega - \omega_0)^2] \tag{SI.61}$$

for all ω, which is easy to verify numerically. To generate the plots in Fig. 2, we use the adaptive quadrature method provided by `mpmath.quad` to compute the frequency-space integrals.

SI.3.4 Frequency suppression in nonlinear networks

In the linear TTI theory derived above, the saddle-point equations decouple across frequencies, with each $C(\omega)$ and $\hat{C}(\omega)$ determined independently by the input and target power spectra at that same frequency ω. This is because the linear activation $\phi(x) = x$ does not mix Fourier modes. As a consequence, $\hat{C}(\omega) \geq 0$ at all frequencies (Eq. (SI.49)), so that learning can only amplify task-relevant frequencies, which is sufficient because the linear activation does not spread power to unwanted frequencies.

The nonlinearity $\phi(\cdot) = \tanh(\cdot)$, by contrast, mixes frequencies together. The pointwise application of $\phi(\cdot)$ in the time domain globally reshapes the power spectrum, producing continuous spectral content with peaks at odd harmonics of the fundamental (as can be seen in Fig. SI.2). As a result, the saddle-point equations for the nonlinear network do not decouple across ω, and the solution at one frequency depends on the solution at all others. This inter-frequency coupling enables a phenomenon with no linear counterpart, namely the suppression of frequencies not required by the task. In the nonlinear DMFT solutions (Fig. SI.2), the conjugate order parameter $\hat{C}(\omega)$ is positive near the target frequency $\omega_\star$, but broadly *negative* elsewhere, suppressing task-irrelevant spectral content. Within this negative region, the odd harmonics $3\omega_\star, 5\omega_\star, \ldots$ appear as local peaks, less suppressed than their surroundings because the nonlinearity inevitably places some power there, but still suppressed relative to baseline. When these frequencies are not part of the target, the associated power does not contribute to performance and should be suppressed.

bioRxiv preprint doi: https://doi.org/10.64898/2026.03.02.708943; this version posted May 18, 2026. The copyright holder for this preprint (which was not certified by peer review) is the author/funder, who has granted bioRxiv a license to display the preprint in perpetuity. It is made available under a CC-BY 4.0 International license.

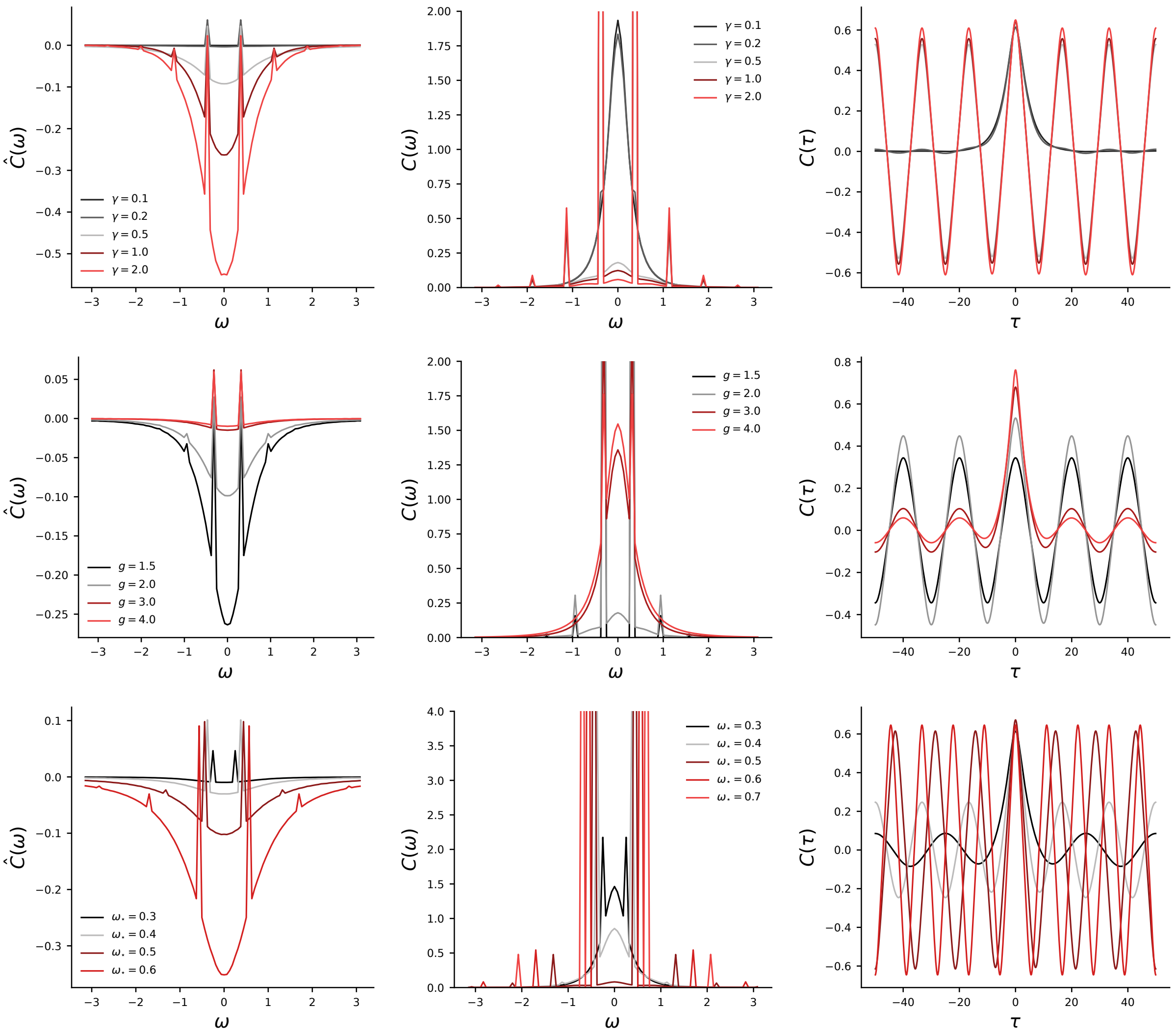

Figure SI.2: Time-translation-invariant DMFT solutions for autonomous ($C^I = 0$) nonlinear RNNs with $\phi(\cdot) = \tanh(\cdot)$, trained to fit a Dirac target power spectrum $C^y(\omega) = \delta(\omega - \omega_\star)$. Columns show, from left to right, the conjugate order parameter $\hat{C}(\omega)$, the power spectrum $C(\omega)$, and the time-domain autocorrelation $C(\tau)$. Each row varies one parameter while holding the other two fixed. Top row: varying γ with $(g, \omega_\star) = (2.5, 0.375)$. Middle row: varying g with $(\gamma, \omega_\star) = (0.5, 0.375)$. Bottom row: varying $\omega_\star$ with $(\gamma, g) = (0.5, 2.5)$. In the left column, $\hat{C}(\omega)$ is positive near the target frequency $\omega_\star$ (amplifying that frequency) and can become negative at other frequencies (suppressing task-irrelevant spectral content, including higher harmonics generated by the nonlinearity).

bioRxiv preprint doi: https://doi.org/10.64898/2026.03.02.708943; this version posted May 18, 2026. The copyright holder for this preprint (which was not certified by peer review) is the author/funder, who has granted bioRxiv a license to display the preprint in perpetuity. It is made available under a CC-BY 4.0 International license.

SI.4 Comparing notions of effective dimensionality of activity

Over the course of this paper, we use two complementary notions of the effective dimensionality of activity. The starting point for both measures is the participation ratio of the finite-size autocorrelation $C_N(t,t')$ over a fixed observation window $[-T/2, T/2]$,

$$\mathrm{PR} = \frac{\left(\int_{-T/2}^{T/2} dt\, C_N(t,t)\right)^2}{\int_{-T/2}^{T/2} dt\, \int_{-T/2}^{T/2} dt'\, C_N(t,t')^2}. \tag{SI.62}$$

Here, we detail how the two measures we use arise from taking the limits $N \to \infty$ and $T \to \infty$ in different orders. What we will find is that if we first take $N \to \infty$ and then $T \to \infty$, the participation ratio is extensive in T, while if we first take $T \to \infty$ and then $N \to \infty$, the participation ratio is extensive in N.

***N* → ∞, then *T* → ∞.** If we take $N \to \infty$ for fixed T and then take $T \to \infty$, all fluctuations in the autocorrelation vanish, and we have $C_N(t,t') \to C(t,t')$ (almost-surely uniformly). Then, suppose that we take $T \gg 1$, such that the system relaxes into a stationary state with $C(t,t') = C(t-t')$. Assuming the contribution of the relaxation timescale to the integral is negligible, we then have

$$\lim_{T\to\infty}\lim_{N\to\infty} \frac{1}{T}\mathrm{PR} = \lim_{T\to\infty} \frac{1}{T} \frac{\left(\int_{-T/2}^{T/2} dt\, C(t,t)\right)^2}{\int_{-T/2}^{T/2} dt\, \int_{-T/2}^{T/2} dt'\, C(t,t')^2} \tag{SI.63}$$

$$= \lim_{T\to\infty} \frac{C(0)^2}{\int_{-T}^{T} d\tau\, \left(1 - \frac{|\tau|}{T}\right) C(\tau)^2} \tag{SI.64}$$

$$= \frac{C(0)^2}{\int_{-\infty}^{\infty} d\tau\, C(\tau)^2}, \tag{SI.65}$$

where we assume that $C(\tau)$ is bounded from above and is square-integrable, and moreover that $|\tau| C(\tau)^2$ is integrable. Using the Fourier transform convention

$$C(\tau) = \int_{-\infty}^{\infty} \frac{d\omega}{2\pi} e^{i\omega\tau} C(\omega), \tag{SI.66}$$

Parseval's theorem implies that

$$\lim_{T\to\infty}\lim_{N\to\infty} \frac{1}{T}\mathrm{PR} = \frac{1}{2\pi} \frac{\left(\int_{-\infty}^{\infty} d\omega\, C(\omega)\right)^2}{\int_{-\infty}^{\infty} d\omega\, C(\omega)^2}. \tag{SI.67}$$

bioRxiv preprint doi: https://doi.org/10.64898/2026.03.02.708943; this version posted May 18, 2026. The copyright holder for this preprint (which was not certified by peer review) is the author/funder, who has granted bioRxiv a license to display the preprint in perpetuity. It is made available under a CC-BY 4.0 International license.

$T \to \infty$, then $N \to \infty$. If $T \gg N$, then we can no longer neglect finite-size fluctuations in C_N. Assuming again that we are nearly in a stationary state, we decompose the finite-size correlation function as

$$C_N(t,t') = C(t-t') + \frac{1}{\sqrt{N}}\Delta_N(t,t'), \tag{SI.68}$$

where $\Delta_N(t,t')$ is an $\mathcal{O}_N(1)$ fluctuation term. Dividing both by T^2, the numerator and denominator of the participation ratio are then

$$\left(\frac{1}{T}\int_{-T/2}^{T/2} dt\, C(t,t)\right)^2 = \left(C(0) + \frac{1}{T\sqrt{N}}\int_{-T/2}^{T/2} dt\,\Delta_N(t,t)\right)^2 \tag{SI.69}$$

and

$$\begin{aligned}\frac{1}{T^2}\int_{-T/2}^{T/2} dt \int_{-T/2}^{T/2} dt'\, C_N(t,t')^2 = \frac{1}{T^2}\int_{-T}^{T} d\tau\,(T-|\tau|)C(\tau)^2 \\ + \frac{2}{T^2\sqrt{N}}\int_{-T/2}^{T/2} dt \int_{-T/2}^{T/2} dt'\, C(t-t')\Delta_N(t,t') \\ + \frac{1}{T^2 N}\int_{-T/2}^{T/2} dt \int_{-T/2}^{T/2} dt'\,\Delta_N(t,t')^2\end{aligned} \tag{SI.70}$$

respectively. We now take $T \to \infty$. The Δ_N-dependent term in the numerator is negligible, as it has zero mean (over realizations) and standard deviation of order $1/\sqrt{NT}$. In the denominator, the contribution of the stationary covariance $C(\tau)^2$ is of order $1/T$ and is thus negligible. The second term in the denominator has mean zero and standard deviation of order $1/(T\sqrt{N})$ assuming that $C(\tau)$ is square-integrable, and is therefore also suppressed. Finally, the third term in the denominator is, up to negligible corrections, the order-one mean-square fluctuation amplitude

$$\Sigma = \lim_{T\to\infty}\frac{1}{T^2}\int_{-T/2}^{T/2} dt \int_{-T/2}^{T/2} dt'\,\langle\Delta_N(t,t')^2\rangle, \tag{SI.71}$$

which is computable as in Clark et al. [26, 91]. This term dominates the denominator, leading to

$$\lim_{N\to\infty}\lim_{T\to\infty}\frac{1}{N}\mathrm{PR} = \frac{C(0)^2}{\Sigma}. \tag{SI.72}$$

bioRxiv preprint doi: https://doi.org/10.64898/2026.03.02.708943; this version posted May 18, 2026. The copyright holder for this preprint (which was not certified by peer review) is the author/funder, who has granted bioRxiv a license to display the preprint in perpetuity. It is made available under a CC-BY 4.0 International license.

SI.5 Sine wave (temporal generalization) task

We trained networks to autonomously generate the two-dimensional sinusoidal target

$$y^\star(t) = \begin{pmatrix} \cos\left(\frac{2\pi t}{T}\right) \\ \sin\left(\frac{2\pi t}{T}\right) \end{pmatrix} \tag{SI.73}$$

with period $T = 10$ (in units of τ), starting from a uniform initial condition $x^0 = 1$ for all neurons, with no external input. We set $\tau = 1$ throughout this task, so all times are dimensionless.

For each (g, γ) configuration, we trained 10 independent networks of size $N = 2500$ via Langevin gradient flow with inverse temperature $\beta = 2000$, step size 0.05, and 75,000 iterations; the Euler integration step was $\Delta t = 0.5$. After training, networks were run autonomously for $5T$ to assess generalization and convergence to limit-cycle dynamics. We identified the onset of nonchaotic behavior by checking whether the normalized autocorrelation returned to unity (threshold 0.98) after $3T$ of autonomous evolution [54]. The DMFT equations were solved numerically on a denser (g, γ) grid using the alternating iteration procedure described in Sec. SI.1.3, with solutions warm-started across adjacent γ values to accelerate convergence. RNN simulations were run on a subset of 9 of the 20 g values; the DMFT was solved on the full grid. All parameters are given in Table SI.1.

SI.6 Reaching task and neural data analysis

We used the dataset from Churchland et al. [6], as reanalyzed by Sussillo et al. [27], consisting of trial-averaged neural and EMG recordings from monkey J performing $B = 27$ reaching movements.

The dataset contains two distinct components. The first is the neural data, namely simultaneously recorded firing rates from 161 neurons in M1/PMd, which we use for the RNN–neural comparisons described below. The second is the RNN training data, consisting of precomputed inputs and target outputs from Sussillo et al. [27]. The inputs are seven-dimensional. The first six are the top principal components of preparatory neural activity, each with the same time-dependent modulation (ramping on during the preparatory period and turning off before movement onset). The seventh is a condition-independent hold cue that is on during the delay and then turns off to trigger the movement. The target outputs are 8-channel EMG signals that have been set to exactly zero until shortly before movement onset.

All data were originally provided at 10 ms resolution. We downsampled by a factor of 2 to a 20 ms time step, yielding 148 time bins for the RNN training data and 118 time bins for the neural recordings, with movement onset at bin 107 and bin 77 respectively. The provided inputs include 8 preparatory delay durations used by Sussillo et al. [27]; we used only zero delay. In the RNN, we set $\tau = 1$ and $\Delta t = 0.4$ in simulation units; because each integration step corresponds to one 20 ms data bin, the physical time

bioRxiv preprint doi: https://doi.org/10.64898/2026.03.02.708943; this version posted May 18, 2026. The copyright holder for this preprint (which was not certified by peer review) is the author/funder, who has granted bioRxiv a license to display the preprint in perpetuity. It is made available under a CC-BY 4.0 International license.

constant is $\tau_{\text{phys}} = (20 \text{ ms})/(\Delta t/\tau) = 50 \text{ ms}$, consistent with the value used by Sussillo et al. [27]. Setting $\tau = 50$ ms with $\Delta t = 20$ ms as reported in the main text and Table SI.2 is equivalent, since only the ratio $\Delta t/\tau$ matters.

EMG targets were shifted to have zero mean during the pre-movement baseline, and both inputs and EMG targets were rescaled to have approximately order-one magnitude when nonzero. To match the initialization conventions of Sussillo et al. [27], who included a factor of $1/\sqrt{D_{\text{in}}} \approx 0.38$ into the input weights at initialization, we further scaled the order-one inputs by a factor of 0.4.

We used $N = 1024$ neurons, compared to $N = 300$ in Sussillo et al. [27], to be in the large-N regime described by the DMFT.

Training was performed via Langevin gradient flow with gradient norm clipping (threshold 10, triggered extremely infrequently). Full parameters are given in Table SI.2.

Equilibration diagnostics. We verified that the Langevin sampler reached equilibrium through several diagnostics. Training MSE curves decrease and plateau before the end of sampling (Fig. 4B), with similar behavior for the full energy (including the Frobenius-norm terms). We also verified that individual weight matrix elements fluctuated on a timescale much shorter than the total sampling time (Fig. SI.3). We note that the MSE continued to decrease slightly when plotted on a log scale, particularly for small g. However, the eigenvalue spectrum of $\frac{g}{\sqrt{N}}\boldsymbol{J}$, which is self-averaging in the large-N limit, showed negligible change over 100,000 iterations deep into sampling (Fig. SI.4), confirming that the macroscopic properties of the network had stabilized.

RNN–neural comparisons. We compared RNN population-level activity to simultaneously recorded M1/PMd firing rates in a ± 400 ms window around movement onset (± 20 time bins at 20 ms resolution). We considered two similarity metrics, centered kernel alignment (CKA) [56] and singular vector canonical correlation analysis (SVCCA) [92] with 10 components; and two preprocessing choices, z-scoring each unit or mean-centering only, yielding four comparisons in total. Sussillo et al. [27] used SVCCA with 10 components. For CKA, we computed the score between the $N \times (B \times n_T)$ RNN activity matrix and the corresponding $161 \times (B \times n_T)$ neural activity matrix, where $n_T = 40$ is the number of time bins in the comparison window; SVCCA was computed analogously. Results are qualitatively similar across all four metric–preprocessing combinations (Fig. SI.5), with an intermediate degree of recurrent restructuring substantially improving the match to neural data relative to the reservoir limit. All four comparisons show clear nonmonotonicity in γ for most values of g, with the exception of CKA without z-scoring.

bioRxiv preprint doi: https://doi.org/10.64898/2026.03.02.708943; this version posted May 18, 2026. The copyright holder for this preprint (which was not certified by peer review) is the author/funder, who has granted bioRxiv a license to display the preprint in perpetuity. It is made available under a CC-BY 4.0 International license.

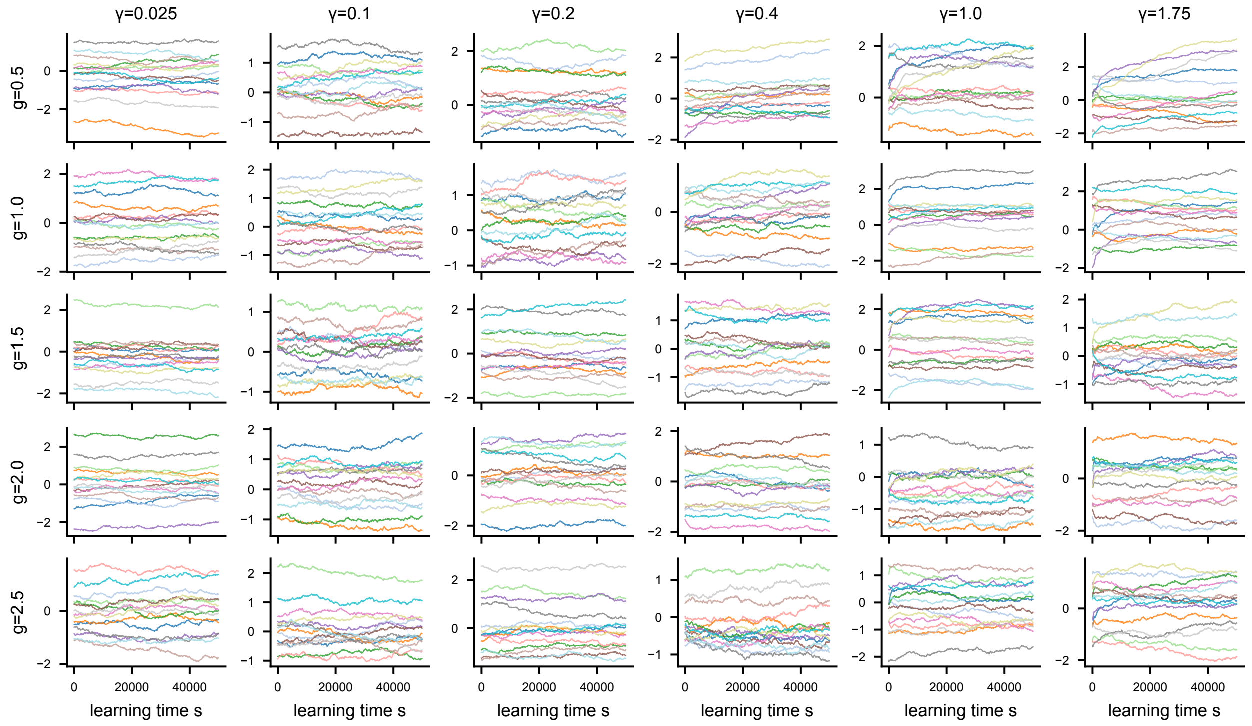

Figure SI.3: **Weight traces during sampling.** Traces of representative elements of J_{ij} over the course of Langevin gradient flow on the reaching task for a grid of g (rows) and γ (columns).

SI.7 p-body generalization of the model

In addition to the RNN with two-body interactions between nodes, we can consider a p-neuron interaction generalization of the dynamics, reminiscent of p-spin models in the spin-glass literature [93]. These models have also gained some attention as descriptions of many-neuron interactions in biology, though the underlying physiology remains a subject of some debate [94]. The dynamics are

$$\frac{d}{dt}x_{i_0}(t) = -x_{i_0}(t) + \frac{g}{N^{p/2}}\sum_{i_1,\ldots,i_p} J_{i_0,i_1,,\ldots,i_p}\phi_{i_1}(t)\cdots\phi_{i_p}(t) + \sum_{a=1}^{D_{in}} U_{ia}I_a(t). \tag{SI.74}$$

Under Langevin dynamics on the tensor $\boldsymbol{J}$, the read-in weights $\boldsymbol{U}$, and the readout weights $\boldsymbol{V}$, we arrive at a structurally identical DMFT in terms of the autocorrelation $C(t,t') = \frac{1}{N}\sum_{i=1}^{N}\phi_i(t)\phi_i(t')$. In the $\beta \to \infty$ limit, the DMFT action takes the form

$$\mathcal{S}(C,\hat{C}) = \frac{\gamma^2}{2}\,\mathrm{tr}\;C^y C^{-1} + \frac{1}{2}\,\mathrm{tr}\,C\hat{C} - \ln W(C,\hat{C}),$$
$$W(C,\hat{C}) = \left\langle \exp\left(\frac{1}{2}\int dt\,dt'\,\phi(t)\,\phi(t')\,\hat{C}(t,t')\right)\right\rangle_{\eta(t)\sim\mathcal{GP}(0,C^I(t,t')+g^2C(t,t')^p)},$$

bioRxiv preprint doi: https://doi.org/10.64898/2026.03.02.708943; this version posted May 18, 2026. The copyright holder for this preprint (which was not certified by peer review) is the author/funder, who has granted bioRxiv a license to display the preprint in perpetuity. It is made available under a CC-BY 4.0 International license.

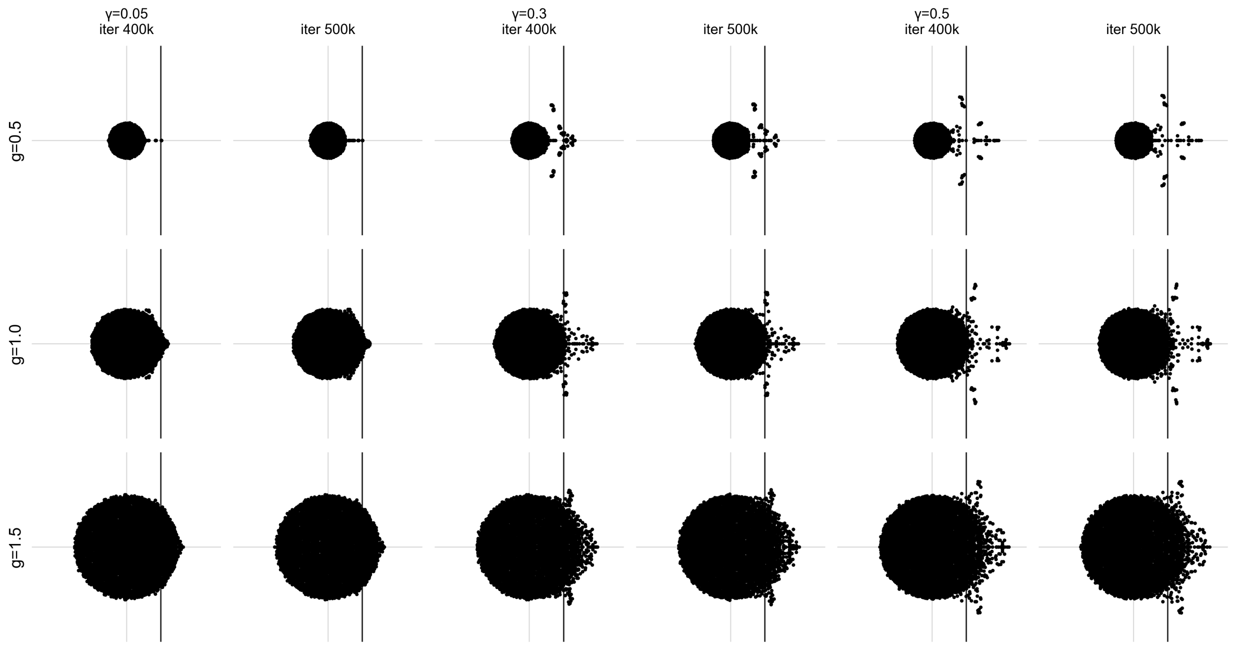

Figure SI.4: **Eigenvalue spectra are stable across sampling.** Eigenvalue spectra of $\frac{g}{\sqrt{N}}\boldsymbol{J}$ at iteration 400,000 (left in each pair) and iteration 500,000 (right), for selected (g, γ) configurations. Rows correspond to g; column pairs correspond to γ. Spectra show negligible change between the two checkpoints.

The main modification relative to the $p = 1$ case is that the noise driving the internal recurrent dynamics $\eta(t)$ has covariance $g^2 C(t, t')^p$ instead of $g^2 C(t, t')$. As before, the $N \to \infty$ limit is determined by the saddle-point equations $\partial \mathcal{S}/\partial C = \frac{\partial \mathcal{S}}{\partial \hat{C}} = 0$.

Nonlinear Dynamics with Linear Activations One advantage of this model is that the dynamics can still be chaotic and autonomous even with linear activations $\phi(x) = x$, provided that $p > 1$. For this choice, the single-site distribution for x remarkably remains Gaussian, even in the feature-learning regime. The DMFT equations for C and $\hat{C}$ therefore close without any single-site average (shown here in the $\beta \to \infty$ limit),

$$\boldsymbol{C} = \left[\boldsymbol{\Sigma}_0^{-1} - \hat{\boldsymbol{C}}\right]^{-1}, \qquad \boldsymbol{\Sigma}_0 = \boldsymbol{C}^I + g^2 \boldsymbol{C}^{\odot p}, \tag{SI.75}$$

$$\hat{\boldsymbol{C}} = \gamma^2 \boldsymbol{C}^{-1} \boldsymbol{C}^y \boldsymbol{C}^{-1} + \left[\boldsymbol{\Sigma}_0^{-1} - \hat{\boldsymbol{C}}\right]^{-1} \cdot \frac{\partial \boldsymbol{\Sigma}_0^{-1}}{\partial \boldsymbol{C}}, \tag{SI.76}$$

where $\boldsymbol{C}^{\odot p}$ denotes the elementwise pth power. This model's DMFT could therefore in principle be solved without resorting to Monte Carlo integration. To maintain stability in the chaotic regime without external drive ($C^I = 0$) and without a saturating nonlinearity, one can constrain the dynamics of the norm of x (restricting to the sphere) [93]. We leave detailed analysis of this model in the feature-learning regime to future work.

bioRxiv preprint doi: https://doi.org/10.64898/2026.03.02.708943; this version posted May 18, 2026. The copyright holder for this preprint (which was not certified by peer review) is the author/funder, who has granted bioRxiv a license to display the preprint in perpetuity. It is made available under a CC-BY 4.0 International license.

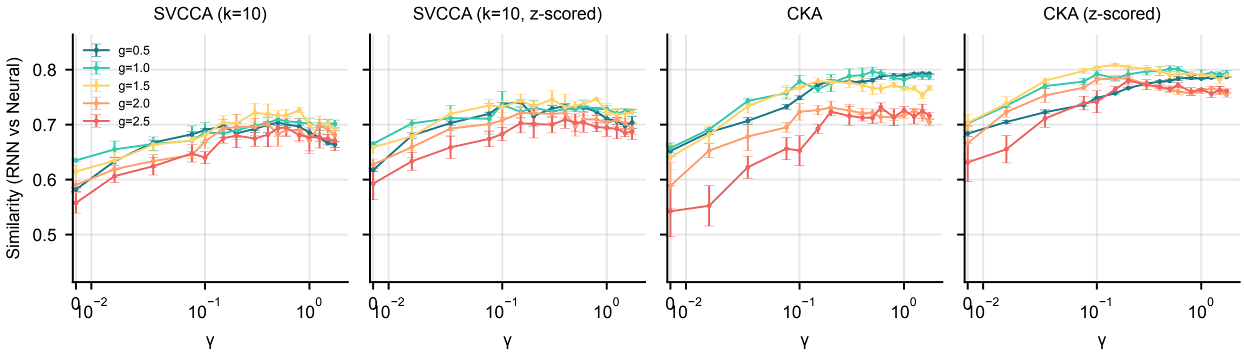

Figure SI.5: **RNN–neural similarity across metrics and z-scoring choices.** Similarity between RNN and M1/PMd population-level activity as a function of γ for different values of g (colors), computed in a ± 400 ms window around movement onset. The rightmost panel is the same as Fig. 5A.

bioRxiv preprint doi: https://doi.org/10.64898/2026.03.02.708943; this version posted May 18, 2026. The copyright holder for this preprint (which was not certified by peer review) is the author/funder, who has granted bioRxiv a license to display the preprint in perpetuity. It is made available under a CC-BY 4.0 International license.

Table SI.1: **Sine wave task parameters.** RNN architecture and Langevin gradient flow training (top), hyperparameter grid (middle), and DMFT numerical solver settings (bottom).

Parameter	Symbol	Value
Architecture		
Neurons	N	2500
Activation function	$\phi(\cdot)$	$\tanh(\cdot)$
Time constant	τ	1 (dimensionless)
Euler step	Δt	0.5
Output dimensions	D_{out}	2 (cos, sin)
External inputs		None
Initial condition	x^0	1.0
Target period	T	10
Langevin gradient flow training		
Inverse temperature	β	2000
Step size		0.05
Training iterations		75,000
Gradient clipping		None
Weight initialization	J_{ij}, V_{ia}	$\mathcal{N}(0,1)$ i.i.d.
Independent runs		10 per (g,γ)
Hyperparameter grid		
Recurrent gain	g	20 values in $[1.0,\ 3.0]$
Output coupling	γ	27 values in $[0,\ 1.2]$ ($g \leq 1.5$);<br>19 values in $[0,\ 1.2]$ ($g > 1.5$)
Trained subset		9 of 20 g values
DMFT solver		
Outer iterations		300
Inner C-update steps		100
MC samples (C update)		10^5
MC samples ($\hat{C}$ update, action gradient)		10^6
Cholesky regularization		10^{-5}
C mixing rate		0.5
$\hat{C}$ mixing rate		0.1
C initialization		Identity
$\hat{C}$ initialization		Zeros
Warm-start across γ		Yes

bioRxiv preprint doi: https://doi.org/10.64898/2026.03.02.708943; this version posted May 18, 2026. The copyright holder for this preprint (which was not certified by peer review) is the author/funder, who has granted bioRxiv a license to display the preprint in perpetuity. It is made available under a CC-BY 4.0 International license.

Table SI.2: **Motor cortex RNN parameters.** Architecture, training, and hyperparameter grid for the reaching task of Sec. 2.6. Each (g, γ) pair was trained with 5 independent random initializations, for a total of 375 networks.

**Parameter**	**Symbol**	**Value**
Architecture		
Neurons	N	1024
Activation function	$\phi(\cdot)$	$\tanh(\cdot)$
Time constant	τ	50 ms
Euler step	Δt	20 ms
Input dimensions	D_{in}	7 (6 temporally-modulated preparatory PCs + 1 go cue)
Output dimensions	D_{out}	8 (EMG activity)
Initial condition	x^0	0.0
Training conditions	B	27 reach directions
Langevin gradient flow training		
Inverse temperature	β	10^6
Step size		0.1
Training iterations		500,000
Gradient clip norm		10
Weight initialization	$J_{ij},\ U_{ia},\ V_{ia}$	$\mathcal{N}(0, 1)$ i.i.d.
Hyperparameter grid		
Recurrent gain	g	$\{0.5,\ 1.0,\ 1.5,\ 2.0,\ 2.5\}$
Output coupling	γ	15 values in $[0.025,\ 1.75]$
Independent runs		5 per (g, γ)
Total networks		375
